# Asymmetric distribution of cytokinins determines root hydrotropism in *Arabidopsis thaliana*

**DOI:** 10.1038/s41422-019-0239-3

**Published:** 2019-10-10

**Authors:** Jinke Chang, Xiaopeng Li, Weihao Fu, Jiawen Wang, Yueyuan Yong, Hongyong Shi, Zhaojun Ding, Hong Kui, Xiaoping Gou, Kai He, Jia Li

**Affiliations:** 1 Ministry of Education Key Laboratory of Cell Activities and Stress Adaptations, School of Life Sciences, Lanzhou University, 730000 Lanzhou, China; 2 The Key Laboratory of Plant Development and Environmental Adaptation Biology, Ministry of Education, College of Life Sciences, Shandong University, Qingdao, 266237 Shandong China

**Keywords:** Plant signalling, Plant molecular biology

## Abstract

The phenomenon of plant root tips sensing moisture gradient in soil and growing towards higher water potential is designated as root hydrotropism, which is critical for plants to survive when water is a limited factor. Molecular mechanisms regulating such a fundamental process, however, are largely unknown. Here we report our identification that cytokinins are key signaling molecules directing root growth orientation in a hydrostimulation (moisture gradient) condition. Lower water potential side of the root tip shows more cytokinin response relative to the higher water potential side. Consequently, two cytokinin downstream type-A response regulators, *ARR16* and *ARR17*, were found to be up-regulated at the lower water potential side, causing increased cell division in the meristem zone, which allows the root to bend towards higher water potential side. Genetic analyses indicated that various cytokinin biosynthesis and signaling mutants, including the *arr16 arr17* double mutant, are significantly less responsive to hydrostimulation. Consistently, treatments with chemical inhibitors interfering with either cytokinin biosynthesis or cell division completely abolished root hydrotropic response. Asymmetrically induced expression of *ARR16* or *ARR17* effectively led to root bending in both wild-type and *miz1*, a previously known hydrotropism-defective mutant. These data demonstrate that asymmetric cytokinin distribution is a primary determinant governing root hydrotropism.

## Introduction

Water distribution in soil is largely heterogeneous.^1^ Accordingly, plants evolved unique capability for their roots to grow towards more water availability in order to absorb sufficient water for survival. Such a phenomenon was first recorded at least 250 years ago in literature and was later named as hydrotropism by Wiesner.^2,3^ Thereafter, various investigations have been conducted trying to elucidate mechanisms controlling root hydrotropism. Using a pea mutant named *ageotropum*, whose roots fail to exhibit both gravitropism and phototropism, Jaffe et al. found that the mutant still possesses normal root hydrotropism, suggesting that root hydrotropism is regulated by a mechanism distinctive from those for gravitropism or phototropism.^4^

Using a loss-of-function genetic approach, Takahashi’s group identified two Arabidopsis point mutants showing reduced root hydrotropism, named as *mizu-kussei 1* (*miz1*) and *miz2*.^5,6^ Map-based cloning indicated that *MIZ1* encodes a protein with an uncharacterized DUF617 domain, designated as the MIZ domain. *miz2* contains a point mutation in GNOM, an endomembrane protein essential for protein trafficking. Using a different screening system, Cassab’s group found a couple of genetic mutants exhibiting root hydrotropic defects. These mutants were named as *no hydrotropic response* (*nhr*) and *altered hydrotropic response* (*ahr1*), respectively.^7,8^ Genes corresponding to the *nhr* and *ahr1* mutants, however, have not been identified.

Although *miz1* showed significantly reduced hydrotropic response, it did not exhibit obvious growth defects under normal laboratory growth conditions, suggesting MIZ1 is quite specific in regulating root hydrotropism.^5^ Consistently, *MIZ1* is predominantly expressed in the root tips of Arabidopsis plants. GNOM, however, is an essential component in protein vesicular trafficking and plays a critical role in a variety of cellular processes.^6^ Within the last decade, significant efforts have been made to understand root hydrotropism via unfolding the biological functions of MIZ1. MIZ1 was found to associate with the cytoplasmic surface of endoplasmic reticulum (ER).^9^ Overexpression of *MIZ1* greatly reduced primary root elongation and lateral root formation largely due to decreased auxin accumulation.^10^ Cytokinin treatment can alter the expression pattern of *MIZ1* at lateral root primordia and suppress their initiation, suggesting MIZ1 is a downstream component in the cytokinin signaling pathway during lateral root development. Recent studies revealed that lateral root occurrence and patterning are largely mediated by moisture gradient.^11^ Higher water potential side of a root produces more lateral roots, which is regulated by SUMO-modification of ARF7. Whether MIZ1 is involved in this process was not discussed. Expression of *MIZ1* in different root tip cell layers suggested that MIZ1 functions mainly in the cortex of transition area between meristem and elongation zones.^12^ More recently, it was found that an asymmetric cytosolic Ca^2+^ signal in the phloem tissue of elongation zone is associated with root hydrotropism.^13^ The Ca^2+^ signal is likely originated from the root cap and slowly moves to the elongation zone where the asymmetric distribution is finally established. In *miz1*, however, long-distance Ca^2+^ transport has been disrupted.

If the moisture gradient is perceived by root caps as supported by numerous pieces of previous evidence, and the elongation zone is where the differential growth can be observed, root meristem sandwiched between root cap and elongation zone should inevitably be involved in the regulation of moisture gradient signal.^4,14–16^ It was not reported, however, the contribution of meristem zone to the root hydrotropic response in the literature. Here we report our discovery that under a hydrostimulation condition, lower water potential side of the root tip shows enhanced cell division activities compared to its counterpart. We demonstrate that such an unequal cell division activity is caused by asymmetric distribution of cytokinins. Lower water potential side of the root tip shows more cytokinin response compared to the higher water potential side. Various cytokinin biosynthesis and signaling mutants show significantly reduced hydrotropic response. Chemical inhibitors that block either cytokinin biosynthesis or cell division can completely disrupt root hydrotropic response. We further discovered that the asymmetric expression of two cytokinin type-A response regulator genes, *ARR16* and *ARR17*, can be induced by hydrostimulation. Asymmetric induction of *ARR16* or *ARR17* is able to promote root bending regardless of the hydrostimulation treatment in Col-0 or *miz1*. These results suggest that plants use different mechanisms for tropic responses. Most of the known tropic responses, such as phototropism and gravitropism, are mainly mediated by asymmetric distribution of auxin, whereas hydrotropism is controlled by the asymmetric distribution of cytokinins.^17–21^ These findings provide conceptual insights into our better understandings of the mechanisms controlling tropic responses in plants.

## Results

### More cell division activities were found at the lower water potential side of the root tip after hydrostimulation treatment

**Fig. 1:**
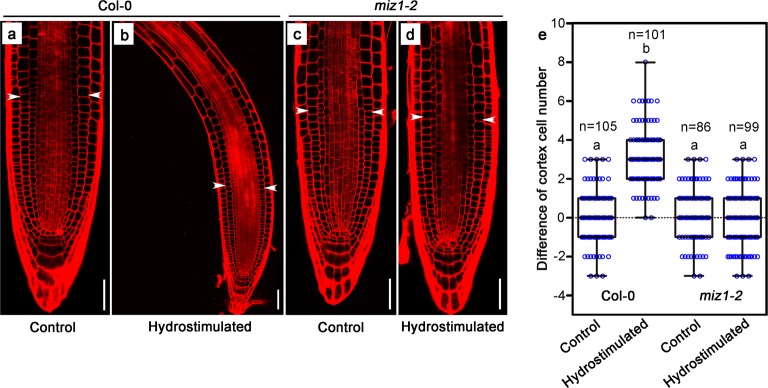
More cell division can be observed at the lower water potential side of the root after hydrostimulation treatment. Propidium iodide-stained root tips of four-day-old wild-type Col-0 seedlings after seedlings were transferred to 1/2 MS split-agar medium (**a**) or hydrostimulating 1/2 MS medium containing 200 mM D-sorbitol at the bottom right side of the Petri dish (**b**) and vertically grown for 2 h. Propidium iodide-stained root tips of four-day-old *miz1-2* seedlings after seedlings were transferred to 1/2 MS split-agar medium (**c**) or hydrostimulating 1/2 MS medium containing 200 mM D-sorbitol at the bottom right side (**d**) and vertically grown for 2 h. **e** Cortex cell number differences of right side versus left side for Col-0 control and *miz1-2* mutant (**a**, **c**), and lower water potential side versus higher water potential side (**b, d**) within a 200 μm meristem zone starting from the quiescent center. White arrow heads in **a**–**d** mark the junction of 200 μm meristem zone from the quiescent center. Each circle represents the measurement of an individual root tip. Boxplots span the first to the third quartiles of the data. Whiskers indicate minimum and maximum values. A line in the box represent the mean. “*n*” represents the number of roots used in the experiment. Scale bars represent 50 µm. One-way ANOVA with Tukey’s multiple comparison test was used for statistical analyses. *P* < 0.001

To examine whether cell division in root meristem zone is involved in hydrotropic response, we compared the root cortex cell numbers between higher water potential and lower water potential sides within a 200-μm meristem region starting from the quiescent center after 4-day-old seedlings were treated with or without hydrostimulation (moisture gradient generated by D-sorbitol) for 2 h. D-sorbitol was widely utilized to generate water potential gradient in laboratory conditions, as described previously (Supplementary information, Fig. S1a–c).^12,13,15,22–24^ We found that in wild-type Col-0 seedlings, the lower water potential side of the roots contain more actively dividing cells and more meristematic cortex cells compared to the higher water potential side (Fig. 1a, b, e, Supplementary information, Fig. S2, Supplementary information, Fig. S3), suggesting uneven cell division activities may have determined root growth direction. Such difference was not observed in untreated wild-type Col-0 or in *miz1-2* mutant (Fig. 1, Supplementary information, Fig. S2), a T-DNA insertional null mutant of *MIZ1*, regardless of the hydrostimulation treatment (Supplementary information, Fig. S1c).^5^

### Asymmetric response of cytokinins was found in wild-type but not in *miz1-2* root tips after hydrostimulation treatment

**Fig. 2:**
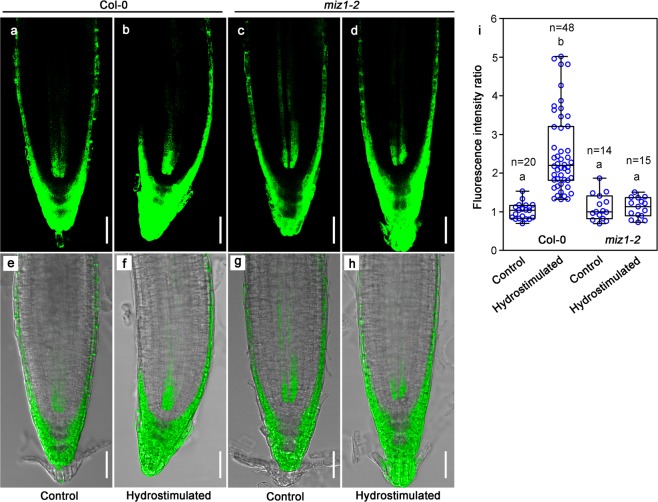
More cytokinin response is observed at the lower water potential side of the root after hydrostimulation treatment. **a–d** Cytokinin response was visualized by a confocal microscope using *TCSn::GFP* transgenic plants. Roots were hydrostimulated for 60 min before the images were taken. In control medium (1/2 MS split-agar medium), levels of GFP signal are similar at both sides of the root tips (**a**, **c**). Lower water potential side showed more GFP signal in the hydrostimulated Col-0 but not *miz1-2* roots (**b, d**). **e–h** GFP signals (**a**–**d**) were merged with their corresponding bright-field pictures. **i** GFP fluorescence ratio of right side versus left side of lateral root caps (controls), or between convex side and concave side of lateral root caps (hydrostimulated seedlings) within a 200-μm meristematic zone starting from the quiescent center. Each circle represents the data from an individual root. Boxplots span the first to the third quartiles of the data. Whiskers indicate minimum and maximum values. A line in the box represents the mean. “*n*” represents the number of roots used in this experiment. Scale bars represent 50 µm. One-way ANOVA with Tukey’s multiple comparison test was used for statistical analyses. *P* < 0.001

If differential cell divisions between two sides of the root tips result in root bending towards higher water potential side, we proposed that some of the plant growth regulators, especially those regulating cell division, might have been unevenly distributed under hydrostimulating condition. Auxin and cytokinins became our initial testing candidates. Many tropic responses such as phototropism and gravitropism are known to be mainly regulated by the asymmetric distribution of auxin.^17–21^ We tested whether auxin shows asymmetric distribution after hydrostimulation treatment and whether auxin redistribution is critical to root hydrotropism. We used transgenic plants carrying *pDR5::GFP*, *pPIN1::PIN1-GFP*, *pPIN2::PIN2-GFP*, *pPIN3::PIN3-GFP* to compare their expression patterns before and after hydrostimulation (Supplementary information, Fig. S4). We found only the GFP signal from *pDR5::GFP*, but not *PINs-GFP*, showed slight asymmetric distribution after hydtrostimulation. The convex side (lower water potential side) accumulated more auxin. In root gravitropic response, usually the concave side of the root accumulates more auxin to repress its growth.^17^ Therefore, the accumulation of auxin at the convex side may play a negative role in root hydrotropic response. Indeed, *pin2*, a T-DNA insertional null mutant of *PIN2*, a mutant lacking root gravitropic response, showed exaggerated hydrotropic response (Supplementary information, Fig. S5). These data are consistent with previous reports, suggesting that auxin is unlikely the major reason for root hydrotropic response in Arabidopsis.^23,25^ We next tested whether asymmetric distribution of cytokinins is the cause of uneven cell division activities and root hydrotropism. We used a previously published *TWO* *COMPONENT* *SIGNALING SENSORnew*::*GREEN FLUORESCENT PROTEIN* (*TCSn*::*GFP*) transgenic plant line (in Col-0 background).^26,27^ We also generated a *miz1-2 TCSn*::*GFP* line by crossing the *TCSn::GFP* line with *miz1-2* and isolating a homozygous line. *TCSn* is a robust and sensitive synthetic promoter and *in vivo* expression of reporter driven by *TCSn* is positively correlated to the levels of bioactive cytokinins or cytokinin response. The promoter activity of *TCSn::GFP* has been widely used to monitor the bioactive levels of cytokinins in roots.^28^ The transgenic seedlings were hydrostimulated on split-agar media for 1 h, the time period after which the root tip bending started to be distinguished in Col-0 seedlings, before analyzing the tissue-level GFP accumulation patterns. Interestingly, we found GFP signal was significantly accumulated at the lower water potential side of the Col-0 root (Fig. 2b, Supplementary information, Fig. S6, Supplementary information, Fig. S7), suggesting more cytokinins accumulated at the lower water potential side. In contrast, such an asymmetric distribution phenomenon was not observed in *miz1-2* roots (Fig. 2d).

### Asymmetric distribution of cytokinins can cause unequal cell division activities and root bending

**Fig. 3:**
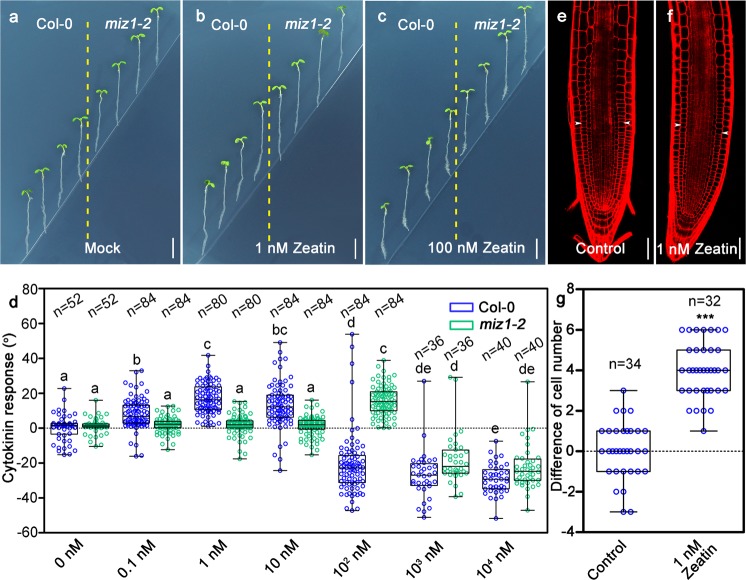
Asymmetric treatment of zeatin alters root growth orientation due to uneven cell division. Root growth curvature after adding 0 nM (**a**), 1 nM (**b**), or 100 nM (**c**) of zeatin at the bottom right side of the split-agar media. **d** Measurements of root growth curvatures after the treatment of different concentrations of zeatin for 24 h. Representative propidium iodide-stained Col-0 root tips showing the root growth curvature without (**e**) or with (**f**) the treatment of 1 nM zeatin at the bottom right side of the media for 2 h. White arrow heads in **e**, **f** mark the junction of 200 μm meristem zone from quiescent center. **g** Cortex cell number differences between right side and left side (control) or zeatin-treated side and untreated side (zeatin treatment) within a 200-μm meristematic region starting from the quiescent center. Each circle represents the measurement of an individual root. Boxplots span the first to the third quartiles of the data. Whiskers indicate minimum and maximum values. A line in the box represents the mean. “*n*” represents the number of roots used in this experiment. Scale bars, for **a**–**c**, represent 5 mm, while for **e–f**, represent 50 µm. One-way ANOVA with Tukey’s multiple comparison test, *P* < 0.001 (**d**), or Student’s *t* test, *P* < 0.0001 (**g**), were used for statistical analyses

To test whether asymmetric distribution of cytokinins is the main reason for root bending after hydrostimulation, we artificially created asymmetric distribution of cytokinins in roots by adding various concentrations of zeatin, one of the bioactive forms of cytokinins, to replace D-sorbitol, at lower right part of the split-agar media (Supplementary information, Fig. S1a).^29^ After 24-hour incubation, Col-0 root tips clearly turned away from 1 nM zeatin medium. Increasing zeatin concentration to 100 nM or greater could allow Col-0 root tips grow toward the zeatin medium (Fig. 3a–c, Supplementary information, Fig. S8). Confocal analysis indicated that low concentrations of zeatin are able to stimulate root tip cell division in Col-0, while high concentrations can repress it (Fig. 3e–g, Supplementary information, Fig. S9, Supplementary information, Fig. S10). Our analysis also revealed that *miz1-2* root tips are less sensitive to the same concentration of zeatin in comparison with Col-0 root tips (Fig. 3a–d). For example, *miz1-2* roots showed no response to 1 nM zeatin treatment, and single side treatment with 100 nM zeatin could make roots to bend away from the treatment, which is opposite to the response of Col-0 roots at the same concentration of zeatin. These data suggest that uneven cell division is caused by asymmetric cytokinin distribution in the root tips after hydrostimulation treatments.

### Type-A response regulators, ARR16 and ARR17, are key components in cytokinin-mediated root hydrotropic response

**Fig. 4:**
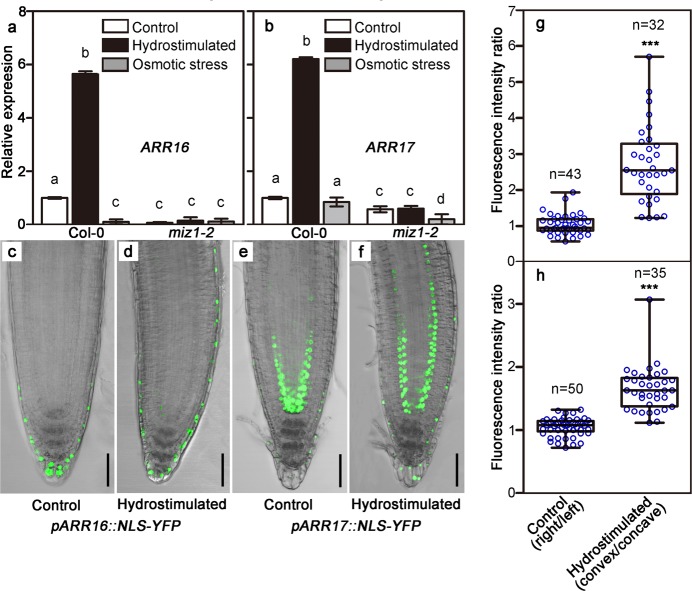
Two type-A response regulator genes of cytokinins, *ARR16* and *ARR17*, can specifically respond to the hydrostimulation treatment. qRT-PCR analyses showing the expression of *ARR16* (**a**), and *ARR17* (**b**) within 0.5 cm root tips can be specifically up-regulated in Col-0 but not in *miz1-2* upon hydrostimulation treatment. Seedlings growing in 1/2 MS medium containing 800 mM D-sorbitol is marked as “osmotic stress” treatment. Data shown represent mean ± SD (*n* = 3). *ACTIN2* was used as an internal control. **c**–**f** Representative merged confocal and bright-field images showing asymmetric expression of *pARR16::NLS-YFP* (**d**), and *pARR17::NLS-YFP* (**f**) can be induced after 60 min hydrostimulation treatments. Their corresponding transgenic plants in Col-0 background were used as controls (**c**, **e**). **g**, **h** Fluorescence intensity ratio of right side versus left side (controls) or convex side versus concave side (hydrostimulated seedlings) of *pARR16::NLS-YFP* (**d**), and *pARR17::NLS-YFP* (**f**) within a 200 μm meristematic region starting from quiescent center. For *pARR16::NLS-YFP* transgenic plants, the measurements were conducted in lateral root caps (**g**), whereas for *pARR17::NLS-YFP* transgenic plants, the measurements were carried out in endodermis cell layers (**h**). Each circle represents the measurement from an individual root. Boxplots span the first to the third quartiles of the data. Whiskers represent minimum and maximum values. A line in the box represents the mean. “*n*” represents the number of roots used in this experiment. Scale bars represent 50 µm. Student’s *t* test, was used for statistical analyses. *P* < 0.0001

If asymmetric distribution of cytokinins is the main factor causing unequal cell division and root bending towards higher water potential region, downstream response regulators of cytokinins should inevitably participate in this process. We first performed qRT-PCR analyses to compare the expression levels of all known cytokinin biosynthesis, catabolism, and type-A and type-B response regulator genes in the root tips of Col-0 and *miz1-2* with or without hydrostimulation (Supplementary information, Fig. S11).^30^ Interestingly, the expression levels of two closely related type-A response regulators, *ARR16* and *ARR17*, showed clear correlation with root hydrotropism. The expression levels of *ARR16* and *ARR17* from the root tips treated on split-agar media with 1/2 MS-1/2 MS or 1/2 MS containing 800 mM sorbitol-1/2 MS containing 800 mM sorbitol are relatively low. Interestingly, these two genes can be significantly up-regulated in root tips upon the treatment of hydrostimulation in Col-0. In addition, their expression levels remained relatively low in *miz1-2* regardless of hydrostimulation (Fig. 4a, b). Previous studies indicated that the transcriptions of type-A response regulators are usually upregulated by cytokinins.^31^ Therefore, we expected that *ARR16* and *ARR17* may also show asymmetric expression patterns in hydrostimulated root tips of Col-0. Indeed, we found in homozygous transgenic seedlings carrying *pARR16::NLS-YFP* or *pARR17::NLS-YFP*, YFP clearly showed asymmetric expression patterns upon the hydrostimulation treatments in Col-0 background (Fig. 4c–h, Supplementary information, Fig. S12, Supplementary information, Fig. S13). In contrast, when these transgenes were introduced into the *miz1-2* background individually by genetic crossing, YFP signals were extremely low in the root tips of the homozygous transgenic lines and asymmetric expression patterns were not noticeable (Supplementary information, Fig. S14). These results indicate that ARR16 and ARR17 are two cytokinin downstream response regulators controlling root hydrotropic response.

### Induced asymmetric expression of *ARR16* or *ARR17* is sufficient to cause root bending in both Col-0 and *miz1-2*

**Fig. 5:**
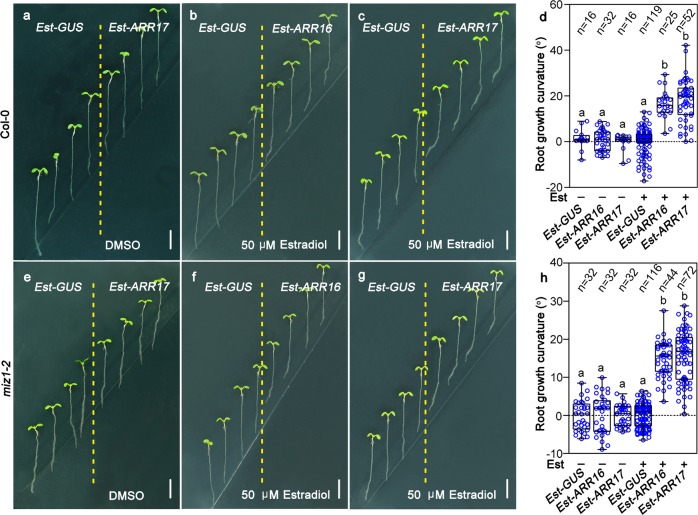
Asymmetrically induced expression of *ARR16* or *ARR17* can alter root growth orientation in both Col-0 and *miz1-2* backgrounds. **a–c** Asymmetric induction of *ARR16* (**b**) or *ARR17* (**c**) in a representative *Est-ARR16* or *Est-ARR17* transgenic line (Col-0 background) by estradiol results in root growing away from the treatment. Solvent DMSO was used as a control (**a**). *Est*-*GUS* transgenic plants were used as vector controls. **d** Measurements of root growth curvatures after induction with DMSO or estradiol as shown in **a**–**c**. **e–g** Asymmetric induction of *ARR16* (**f**) or *ARR17* (**g**) in a representative *Est-ARR16* or *Est-ARR17* transgenic line (*miz1-*2 background) by estradiol also can make the roots to grow away from the treatment. **h** Measurements of root growth curvatures after induction with DMSO or estradiol as shown in **e–g**. Each circle represents the measurement from an individual root. Boxplots span the first to the third quartiles of the data. Whiskers represent minimum and maximum values. A line in the box represents the mean. “*n*” represents the number of roots used in this experiment. Scale bars represent 5 mm. One-way ANOVA with Tukey’s multiple comparison test was used for statistical analyses, with *P* < 0.001

Our results indicated that hydrostimulation can induce asymmetric accumulation of cytokinins, causing more expression of *ARR16* and *ARR17* at the lower water potential side. To test whether asymmetric expression of *ARR16* or *ARR17* is enough to lead root bending, we first generated a number of transgenic plants, including estradiol inducible *ARR16* and *ARR17* (*Est-ARR16* and *Est-ARR17*) and vector control *Est-GUS* in Col-0 and *miz1-2* backgrounds. We first confirmed that the asymmetric expression of target genes can be effectively induced by estradiol (Supplementary information, Fig. S15). One highly inducible representative homozygous line from each transgenic event was selected for further analyses. Our data indicated that asymmetric induction of *ARR16* or *ARR17* in either Col-0 or *miz1-2* background by estradiol can effectively induce uneven cell division, resulting in bending away of the root tips from the induced side (Fig. 5, Supplementary information, Fig. S16). On the other hand, roots of the transgenic plants carrying *Est-GUS* showed no bending regardless of estradiol induction. These results indicate that the asymmetric expression of *ARR16* and *ARR17* can effectively direct root bending during hydrotropic response.

### Various cytokinin biosynthesis and signaling mutants showed significantly reduced root hydrotropic response

**Fig. 6:**
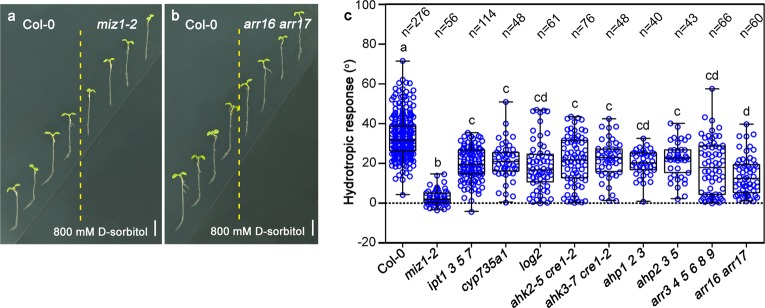
Cytokinin biosynthesis and signaling mutants showed decreased responses to hydrostimulation treatments. Hydrotropic responses of representative wide-type (Col-0), *miz1-2* (**a**) and *arr16 arr17* (**b**) double mutant. **c** Measurements of root growth curvatures of cytokinins-related mutants in response to hydrostimulation treatments. Each circle represents the measurement from an individual root. Boxplots span the first to the third quartiles of the data. Whiskers represent minimum and maximum values. A line in the box represents the mean. “*n*” represents the number of roots used in this experiment. Scale bars represent 5 mm. One-way ANOVA with Tukey’s multiple comparison test was used for statistical analyses. *P* < 0.001

If cytokinins play key roles in regulating root hydrotropic response, we expected that the mutants with either cytokinin biosynthesis or signal transduction blocked should show altered root hydrotropism response. Because complete block of the biosynthesis or signal transduction of cytokinins usually leads to rootless or lethality phenotypes, we tested the root hydrotropic response of a number of known Arabidopsis partially loss-of-functional biosynthesis mutants including *ipt1 3 5 7*, *cyp735a1*, *log2*, and several partially loss-of-functional signaling mutants such as *ahk2-5 cre1-2*, *ahk3-7 cre1-2*, *ahp1 2 3*, *ahp2 3 5*, *arr3 4 5 6 8 9*, and *arr16 arr17*.^32–36^ All these mutants showed drastically decreased cytokinin signaling output and cell division activities in their root meristem zones (Supplementary information, Fig. S17). Compared to Col-0, these mutants displayed significantly reduced root hydrotropism (Fig. 6). To further confirm the significance of cytokinins and cell division in root hydrotropism, we used two well-documented chemical inhibitors. We employed lovastatin to interfere with cytokinin biosynthesis and colchicine to inhibit cell division (Supplementary information, Fig. S18).^30,37–40^ After pretreated with lovastatin or colchicine for one day to deplete endogenous cytokinins or to reduce cell division activities, the seedlings were then transferred to hydrostimulating medium containing either lovastatin or colchicine. Interestingly, with the presence of any one of the inhibitors, root hydrotropism was completely diminished, although roots were still able to elongate possibly due to cell expansion (Supplementary information, Fig. S19). We gravistimulated the same sets of plants by turning the petri dishes some degrees to make root tips horizontally placed and incubated them for additional 24 h. All the plant roots exhibited normal gravitropic responses (Supplementary information, Fig. S20). These genetic and chemical analyses demonstrate that cytokinins-mediated cell division is indeed the key factor for root hydrotropism.

## Discussion

**Fig. 7:**
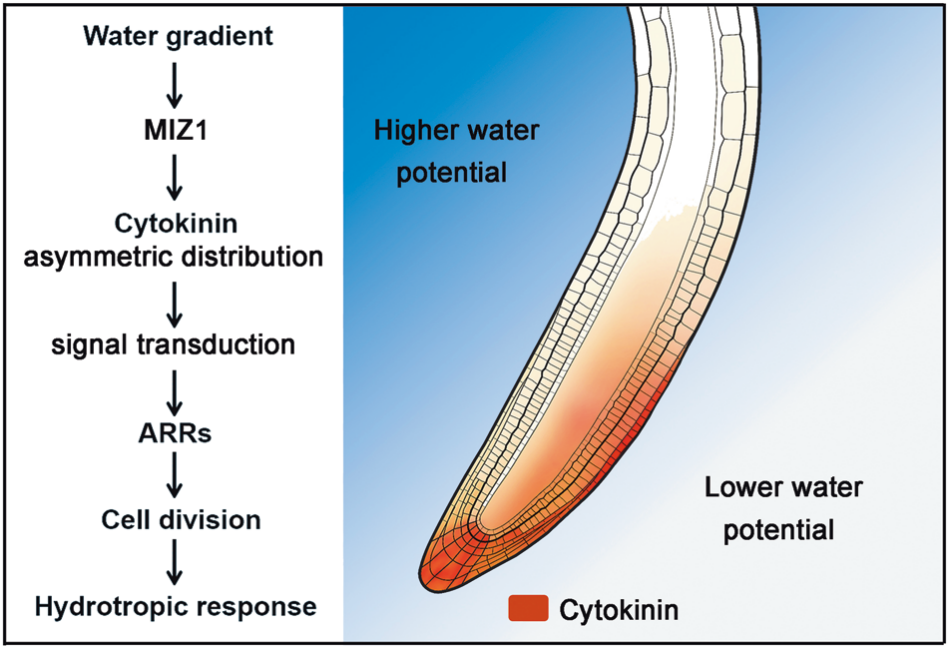
A current model explaining how root hydrotropism is achieved. When roots are growing in an environment with unequal water potential, cytokinins are accumulated at the lower water potential side, up-regulating the expression of *ARR*s such as *ARR16* and *ARR17*, causing more cell division at the lower water potential side of the root tip. As a consequence, the root tip bends towards the higher water potential side of the root. MIZ1 is required for the formation of cytokinin asymmetric distribution and the expression levels of *ARR*s

Numerous early experiments suggested that the root cap is essential for sensing water gradient. Removal of the root caps or covering the root caps with hydrophobic material resulted in roots insensitive to hydrostimulation treatment.^4,14,41,42^ Molecular mechanisms governing root hydrotropic response, however, are largely unrevealed. We hypothesized that the meristem zone adjacent to the root cap may play an indispensable role in regulating root growth orientation in heterogeneous moisture distribution environment. As a matter of fact, we found uneven cell division in the meristem zone is a major reason causing root bending towards higher water potential side. This process mainly depends on the asymmetric distribution of cytokinins. Earlier studies demonstrated that the more cells produced in the meristem per time, the more cells flow into the elongation zone within the same time period, causing quicker elongation.^43^ Although faster growth appears at the elongation zone of the lower water potential side after hydrostimulation treatment, we found the differential growth actually starts from the root meristem zone. Blocking cell division in the meristem zone can completely destroy root hydrotropic response. We also found that asymmetric distribution or response of cytokinins occurs mainly in the meristem zone after hydrostimulation treatment. Reducing the biosynthesis of cytokinins in the meristem zone by lovastatin treatment resulted in the roots irresponsive to hydrostimulation. Consistently, cytokinin biosynthetic mutants showed significantly reduced hydrotropic response. These results demonstrate that asymmetric distribution of cytokinins in the meristematic zone is the key for uneven cell division and subsequent root hydrotropism (Fig. 7).

In Arabidopsis, cytokinins are perceived by membrane-localized histidine kinase receptors including AHK2, AHK3, and AHK4/CRE1/WOL and transduced via a His-Asp phosphorelay to activate response regulators and transcription factors in the nucleus to regulate various processes in plant growth, development, and stress adaptations.^44,45^ Three groups of functionally distinctive response regulators were found as downstream cytokinin signaling components in Arabidopsis.^46^ Type-B, but not type-A or type-C response regulators serve as transcription factors directly regulating the expression of Type-A genes.^47^ Therefore the expression levels of type-A response regulators are usually correlated to the bioactive levels of cytokinins. Previous studies found that transgenic Arabidopsis plants overexpressing type-A genes showed elongated primary roots.^48^ On the other hand, high order type-A response regulator mutants showed reduced meristem cell numbers and decreased primary root length.^36,49^ We also found *arr16 arr17*, a type-A double mutant, showed significantly reduced meristematic cortex cell number (Supplementary information, Fig. S17). The functions of type-C response regulators, however, are not well characterized.

Numerous evidence indicated that the biological effects of cytokinins on cell division are dosage dependent. Cytokinins were thought to have opposite roles in regulating shoot and root apical meristem development. When *CYTOKININ OXIDASEs* (*CKXs*) were overexpressed, shoot apical meristem was found to show diminished activity, whereas the cell division activity in root apical meristem was significantly enhanced.^50^ GUS staining using *pARR5:GUS* transgenic plants indicated that overexpression of *CKX1* can almost entirely eliminate cytokinin response in the shoots, but can only partially reduce cytokinin response in roots. Previous analyses indicated that exogenous application of low concentration of cytokinins can enhance root growth, whereas elevated amount of cytokinins can greatly inhibit root growth.^48^ These data suggest that different plant organs/tissues possess different sensitivities to cytokinins. Roots appear to be more sensitive to the treatment of cytokinins than shoots. It is possible that *CKX1* overexpression only can reduce cytokinins from an inhibitory level to the level which can promote cell division. When 1 nM zeatin was exogenously applied at bottom right side of the split-agar medium, the root side near zeatin showed increased cell division and grew away from zeatin. When exogenous zeatin concentration was increased to 100 nM or more, the root tips grew towards zeatin (Fig. 3a–c). Our observation indicates that high concentration of cytokinins inhibits cell division and root growth, whereas low concentration of cytokinins promotes cell division and root elongation.

We used a *TCSn::GFP* transgenic line to detect the endogenous bioactive levels of cytokinins in Arabidopsis. Because *TCSn::GFP* is a transcriptional reporter system, we cannot completely rule out the possibility that other factors affecting the cytokinin signaling may also regulate the expression of *TCSn::GFP*.^26^ But our genetic and physiological data strongly suggest the asymmetric distribution of cytokinins is a main factor leading to the hydrotropic response. After hydrostimulation treatment, we found more GFP signals were accumulated in the lateral root cap cells at the lower water potential side of the root. We could not detect the GFP signal in the cortex. The concentration of cytokinins in cortex may be too low to be detected by *TCSn::GFP*.^51^ It is highly possible that the asymmetric distribution is also present in the cortex, endodermis, and the vascular tissues. Several of our observations support such a scenario. For example, after hydrostimulation, lower water potential side of the root showed more cell number in the cortex compared to the higher water potential side (Fig. 1). In addition, one of the type-A response regulators, *ARR17*, showed higher expression level in the endodermis cell layer adjacent to the lower water potential side of the root (Fig. 4). Finally, a chemical inhibitor, lovastatin, which can effectively suppress cytokinin biosynthesis, can significantly decrease the expression levels of *ARR16* and *ARR17*, inhibit cell division in root meristem, and completely abolish root hydrotropism (Supplementary information, Fig. S18, Supplementary information, Fig. S19, Supplementary information, Fig. S20). These results strongly support that asymmetric distribution of cytokinins beyond the observed lateral root cap is also critical for determining root hydrotropism.

During an on-gel assay, we observed that cytokinin response at the higher water potential side was rapidly decreased, which is ahead of the gradually increased cytokinin response at the lower water potential side (Supplemental information Fig. S7). This result suggests that uneven cytokinin distribution is likely the consequence of both uneven degradation and biosynthesis of cytokinins in the root tips. We found the uneven distribution or response of cytokinins is partially dependent on the synthesis of new proteins, as the cytokinins asymmetric distribution or response was significantly decreased after hydrostimulation in the presence of cykloheximide (CHX), an eukaryote protein synthesis inhibitor (Supplementary information, Fig. S21). Of course, we cannot completely rule out the possible contribution of horizontal cytokinin transport in the root meristem zone. Possible roles of cytokinins in regulating hydrotropism response were discussed in a previous report.^8^ It was found that 300 nM kinetin, an adenine type of cytokinins, can effectively recover hydrotropism defects of *ahr1* and *nhr1* mutants. Since the corresponding genes of *ahr1* and *nhr1* are not known, the molecular mechanisms have not been elucidated.

Early genetic analysis indicated that MIZ1 specifically regulates root hydrotropism in Arabidopsis.^5^ The detailed molecular mechanisms, however, are not well understood. Our analyses indicated that the asymmetric distribution of cytokinins relies on a functional MIZ1. We also found the expression levels of *ARR16* and *ARR17* in *miz1-2* remained relatively low regardless of hydrostimulation treatments. One side expression of either *ARR16* or *ARR17* can effectively induce *miz1-2* root tip bending. Our results therefore suggest MIZ1 is somehow involved in mediating both cytokinin redistribution and signaling during root hydrotropic response. Our investigation also indicated that the expression patterns of *MIZ1* are not regulated by hydrostimulation or exogenous application of cytokinins (Supplementary information, Fig. S22).

Identification of cytokinins in controlling root hydrotropism contributes to our better understanding of how plants adapt to ever-changing environments. Many questions, however, remain to be investigated in the future. For example, what is the sensor for perceiving moisture gradient signal and how does MIZ1 regulate cytokinin redistribution and signaling in roots? What is the relationship between asymmetric distribution of cytokinins in meristematic zone and long-distance transported Ca^2+^ in the elongation zone? Our preliminary data suggest that Ca^2+^ is not required for the asymmetric cytokinins distribution or response (Supplementary information, Fig. S23). Our on-gel analysis revealed that the asymmetric response of cytokinins can be observed as early as 20–30 min after hydrostimulation treatment (Supplementary information, Fig. S7, Supplementary information, Fig. S13, Supplementary information, Fig. S24). However, the asymmetric distribution of Ca^2+^ can be seen in the elongation zone about 60 min after the treatment based on the previous report.^13^ Whether asymmetric cytokinins distribution or response leads to the asymmetric distribution of Ca^2+^ is an outstanding question to be explored in the future. Uncovering the detailed molecular mechanisms via which root hydrotropism is controlled can help us to carry out novel strategies to generate commercially important plants with higher efficiency of water usage, better chance of survival, and higher productivity under drought environmental conditions.

## Materials and Methods

### Plant material, growth conditions and root hydrotropism assays

All plants used for various treatments (except lovastatin and colchicine treatments) were 4-day-old *Arabidopsis thaliana*. Columbia accession (Col-0) was used as wild-type. *miz1-2* (SALK_076560), *cyp735a1* (SALK_093028), and *log2* (SALK_052856) were obtained from the Arabidopsis Biological Resource Center (ABRC). Double or high order mutants including *ipt1 3 5 7*, *ahk2-5 cre1-2*, *ahk3-7 cre1-2*, *ahp1 2 3*, *ahp 2 3 5*, and *arr3 4 5 6 8 9* were provided by professors Guangqin Guo and Yurong Bi (Lanzhou University, China).^33–36^ *arr16*, *arr17*, and *arr16 arr17* mutants were obtained from Jianru Zuo (Institute of Genetics and Developmental Biology, Chinese Academy of Sciences). *pin2* (SALK_144447) mutant was provided by professor Jianwei Pan (Lanzhou University, China). *TCSn::GFP* transgenic plants in Col-0 was provided by professor Zhaojun Ding (Shandong University, China) and professor Bruno Müller (University of Zurich, Switzerland). Binary constructs *pMIZ1::GUS*, *pARR16::NLS-YFP* and *pARR17::NLS-YFP* were generated by introducing the corresponding promoters to our modified gateway constructs *pBIB-BASTA-GUS-GWR* or *pBIB-BASTA-NLS-YFP-GWR*. The *pDR5::GFP*, *pPIN1::PIN1-GFP*, *pPIN2::PIN2-GFP*, and *pPIN3::PIN3-GFP* transgenic plants were obtained from professors Guangqin Guo (Lanzhou University, China). A gateway construct of estradiol inducible expression system, *pER8-GWR*, was modified from *pER8*.^52^ Primer information used for mutant genotyping and DNA cloning can be identified in supplementary information, Table S1 and supplementary information, Table S2.

Surface-sterilized Arabidopsis seeds were kept in a 4 °C refrigerator for 2 days for a stratification treatment, and geminated vertically on half-strength Murashige and Skoog (1/2 MS) agar (1% w/v) plates supplemented with 1% sucrose (w/v) in a 22 °C and 16 h light/ 8 h dark growth chamber.

Hydrotropism assays were performed as shown in Supplementary information, Fig. S1 using 4-day-old seedlings in a split-agar system modified from previously reports.^22,24^ We added 200 mM D-sorbitol at the bottom right side of the Petri dish if the roots were used for propidium iodide staining after hydrostimulation treatment. In other experiments, 800 mM D-sorbitol was used.

On-gel hydrostimulation analyses were carried out by using a concave slide. 1/2 MS with agar medium was pulled into the pit of the slides. After the medium was solidified, half of medium was removed and replaced with the same medium but containing additional 800 mM D-sorbitol. Root tips were placed about 2–3 mm from the border of the media for hydrostimulation treatment. The root tips were continuously imaged using a confocal laser scanning microscope (Leica TCS SP8) with a 20-min interval.

Before investigating gene expression patterns, such as *TCSn::GFP*, *pCYCB1;1::GUS*, *pARR16::NLS-YFP* or *pARR17::NLS-YFP*, roots were hydrostimulated for 60 min. If the roots were used for counting cell numbers after propidium iodide staining, the time of hydrostimulation was 120 min. To test gene transcription levels, the roots were hydrostimulated for 16 h. 5 mm root tips were collected for total RNA extraction and qRT-PCR analyses.

### Root orientation determination during imaging

It is important to keep the orientation of roots unchanged during imaging. For wild-type seedlings, after hydrostimulation, the orientation of most of the roots can be distinguished under a dissect microscope. Usually, lower water potential side of the root shows an obvious outgrown phenotype compared to the higher water potential side. After transferring the seedlings to the slide with or without staining, the original orientation can be easily retrieved based on the morphology of the roots. For confocal analysis, the orientation can be kept unchanged during transferring the seedlings from the media to slides by parallel moving with flat twisters under the dissect microscope.

### EdU staining

Four-day-old seedlings were transferred from 1/2 MS supplemented with 1% agar (w/v) and 1% sucrose (w/v) medium to split-agar hydrostimulating medium with 800 mM D-sorbitol at bottom right side and 10 μM EdU in both sides or to normal 1/2 MS medium with different concentrations of zeatin containing 10 μM EdU. For controls, seedlings were transferred to split-agar medium plates without D-sorbitol or normal 1/2 MS medium without zeatin but with 10 μM EdU in both sides. After 60 min incubation, seedlings were washed in 1/2 MS liquid medium (3 × 5 min) supplemented with 1% sucrose (w/v) to remove excessive EdU. EdU detection was performed after washing the seedlings in phosphate-buffer saline (PBS, pH 7.4) containing 0.5% (v/v) triton X-100 (2 × 5 min). The EdU detection cocktail was made according to the protocol from Click-iT^®^ EdU imaging Kit (Invitrogen). The fluorochrome used was Alexa Fluor^®^ 488. Samples were submerged into the cocktail for 30 min in the dark, followed by washing in PBS (pH 7.4, 3 × 5 min). Samples were then covered by PBS (pH 7.4) containing 8 µg/ml Hoechst 33342 (component G of Click-iT^®^ EdU imaging Kit, Invitrogen) and incubated for 30 min in the dark, followed by washing in PBS (pH 7.4, 3 × 20 min). The root tips ware imaged using a confocal laser scanning microscope (Leica TCS SP8). The fluorescence signals were measured by the imaging software.

### GUS staining

The GUS staining protocol was modified from a previous report.^53^ Four-day-old homozygous transgenic seedlings harboring *pCYCB1;1::GUS* or *pMIZ1::GUS* were transferred from normal 1/2 MS medium supplemented with 1% agar (w/v) and 1% sucrose (w/v), to the plates without (control) or with 800 mM D-sorbitol. After 60 min treatment, seedlings were then transferred to ice-incubated 90% acetone and incubated for 15 min. Acetone was then removed completely and rinse solution (50 mM sodium phosphate buffer pH 7.2, 0.5 mM K_3_Fe(CN)_6,_ 0.5 mM K_4_Fe(CN)_6_) was added and kept at room temperature for 5 min. Rinse solution was then removed completely and staining solution (the rinse solution plus 2 mM X-gluc) was added and incubated at 37 °C in the dark. The staining time was 30 min for *pCYCB1;1::GUS* transgenic seedlings and 12 h for *pMIZ1::GUS* transgenic seedlings. The samples were then washed with ethanol series, 15%, 30%, 50%, 70%, 80%, 95%, 100 and 85% for 30 min each at room temperature. The samples were then kept in 70% ethanol overnight. Ethanol was removed and replaced with chloral hydrate solution (80% chloral hydrate solution (w/v), 20% glycerin (v/v), and 20% purified water (v/v)). After 48 h, root tips were imaged. The GUS staining was measured according to a previously reported protocol.^54^

### Cytokinin treatment

For the cytokinin inducing root bending experiment, four-day-old seedlings were transferred from the 1/2 MS medium supplemented with 1% (w/v) agar and 1% sucrose (w/v), to the split-agar system with different concentrations of zeatin (*trans*-zeatin) at the bottom right side of the medium. The roots were propidium iodide stained and the confocal images were taken after 120 min treatment, whereas the regular root images were taken after one day. For detection of gene expression responding to cytokinins, four-day-old seedlings were transferred from 1/2 MS with 1% agar and 1% sucrose (w/v) medium to the same 1/2 MS medium containing different concentrations of zeatin. After 16 h, 5 mm root tips were collected for total RNA extraction and qRT-PCR analyses.

### qRT-PCR

Four-day-old seedlings were transferred from 1/2 MS media supplemented with 1% agar (w/v) and 1% sucrose (w/v), to the hydrostimulating medium with 800 mM D-sorbitol at the bottom right side, or to the osmotic stress medium (1/2 MS medium supplemented with 1% agar and 1% sucrose (w/v) containing 800 mM D-sorbitol), 5-mm-long root tips were collected after 16 h for total RNA extraction and qRT-PCR analysis. The sequences of primers used for qRT-PCR were listed in Supplementary information, Table S3.

### Estradiol-induced gene expression

Four-day-old seedlings containing *Est-GUS*, *Est-ARR16* or *Est-ARR17* inducible constructs, were transferred from 1/2 MS medium to estradiol inducible medium (similar to the hydrostimulating medium, but replaced D-sorbitol with 50 µM estradiol), induced for 2 h for investigation of cell number or GUS expression pattern between estradiol-treated and untreated side of the root. For studying root growth orientation, the induction time period is one day.

### Lovastatin, colchicine, or BAPTA-AM treatment

For hydrotropism and gravitropism analyses, three-day-old seedlings were transferred from 1/2 MS medium supplemented with 1% agar (w/v) and 1% sucrose (w/v) to the same medium but containing 50 nM lovastatin, 100 µM colchicine, or 10 µM BAPTA-AM and pretreated for one day (for controls, the same volumes of DMSO as the solvent of the inhibitors were added). The seedlings were transferred to hydrostimulating medium containing 800 mM D-sorbitol at the bottom right side and the same concentration of lovastatin, colchicine, or BAPTA-AM at both sides, cultured for additional two days. After photographed, the Petri dish with medium and seedlings were rotated clockwise to make root tips horizontally placed and cultured for one additional day. The root growth curvatures for hydrotropism and gravitropism were measured using Image J.

### Fluorescence intensity measurement

To count the cortex cell numbers at the meristematic region, root tips stained with propidium iodide were imaged by a Leica confocal laser scanning microscope, then the fluorescence intensity was measured using Leica software quantification tools. Within 200 µm above the quiescent center, each of the fluorescence peak in the cortex indicates a cell wall. For *TCSn::GFP*, *pARR16::NLS-YFP* the fluorescence intensities were measured by linear average in the lateral root caps within 200 µm above the quiescent center. For *pARR17::NLS-YFP* the fluorescence intensities were measured by linear average within endodermis cells (Supplementary information, Fig. S25). For the relative root GFP intensity analyses from EdU staining in *pDR5::GFP*, *pPIN1::PIN1-GFP*, *pPIN2::PIN2-GFP*, *pPIN3::PIN3-GFP* transgenic plants, the measured areas are marked in each of the figures presented.

### Statistical analysis

Data analyses in our research were performed using both Student’s *t*-test and one-way ANOVA test. The Student’s *t*-test was employed to determine if two data sets have significant differences. “***” indicates a significant difference and “ns” means no significant difference between two data sets based on the Student *t*-test with the *P* value less than 0.0001.

ANOVA was used when there were more than two data sets to compare. One-way ANOVA with Tukey’s multiple comparison test was applied at the *P*-value 0.001 significance level to each data set. Letters were used to indicate significance levels between all the data sets. Same letter indicates that there were no significant differences. Whereas, different letters indicate that there were significant differences between the data sets.

## Supplementary information

Supplementary information, Figure S1

Supplementary information, Figure S2

Supplementary information, Figure S3

Supplementary information, Figure S4

Supplementary information, Figure S5

Supplementary information, Figure S6

Supplementary information, Figure S7

Supplementary information, Figure S8

Supplementary information, Figure S9

Supplementary information, Figure S10

Supplementary information, Figure S11

Supplementary information, Figure S12

Supplementary information, Figure S13

Supplementary information, Figure S14

Supplementary information, Figure S15

Supplementary information, Figure S16

Supplementary information, Figure S17

Supplementary information, Figure S18

Supplementary information, Figure S19

Supplementary information, Figure S20

Supplementary information, Figure S21

Supplementary information, Figure S22

Supplementary information, Figure S23

Supplementary information, Figure S24

Supplementary information, Figure S25

Supplementary information, Table S1

Supplementary information, Table S2

Supplementary information, Table S3
