# Supplementary material for: Asymmetric distribution of cytokinins determines root hydrotropism in Arabidopsis thaliana
Source: Cell Res. 2019 Oct 10;29(12):984–93. doi: 10.1038/s41422-019-0239-3 (PMC6951336; doi:10.1038/s41422-019-0239-3)
Supplement: Supplementary file 1 — Supplementary information, Figure S1 [file 41422_2019_239_MOESM1_ESM.pdf]

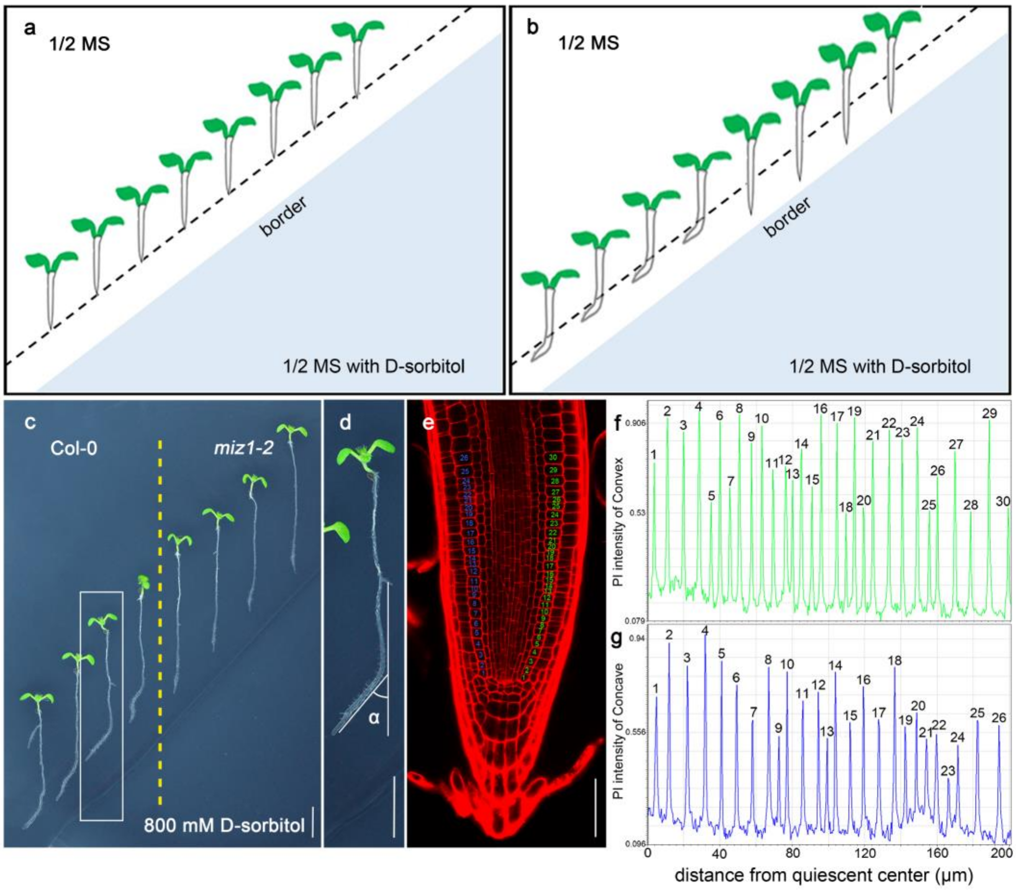

Supplementary information, Fig. S1 Approaches used for hydrostimulation treatment, root bending measurement, and meristem cortex cell number counting. a-d, 1/2 MS medium supplemented with 1% sucrose and 1% agar (w/v) was pulled in a Petri dish. After solidified, right bottom half of the medium was removed and replaced with 1/2 MS medium supplemented with 1% sucrose, 1% agar (w/v), and 200 or 800 mM D-sorbitol. Such a medium is named as hydrostimulating (moisture gradient) medium. To perform hydrostimulating analysis, four-day-old seedlings were transferred to the hydrostimulating medium with root tips 0.5 cm away from the border (**a**), seedlings were then vertically grown on the medium for specified time periods (**b**), photographed (**c**), and root growth curvatures were measured by Image J (**d**). **e-g**, cell numbers were counted by the peaks of cell wall florescence by using a Leica software after propidium iodide (PI) staining. For example, in a root showing in **e**, convex side contains 30 cortex cells (**f**), and concave side contains 26 cortex cells (**g**) within a 200 μm meristematic zone starting from the quiescent center. Scale bars in (**c**) and (**d**) represent 5 mm. Scale bar in (**e**) represents 50 μm.
