# Supplementary material for: Asymmetric distribution of cytokinins determines root hydrotropism in Arabidopsis thaliana
Source: Cell Res. 2019 Oct 10;29(12):984–93. doi: 10.1038/s41422-019-0239-3 (PMC6951336; doi:10.1038/s41422-019-0239-3)
Supplement: Supplementary file 2 — Supplementary information, Figure S2 [file 41422_2019_239_MOESM2_ESM.pdf]

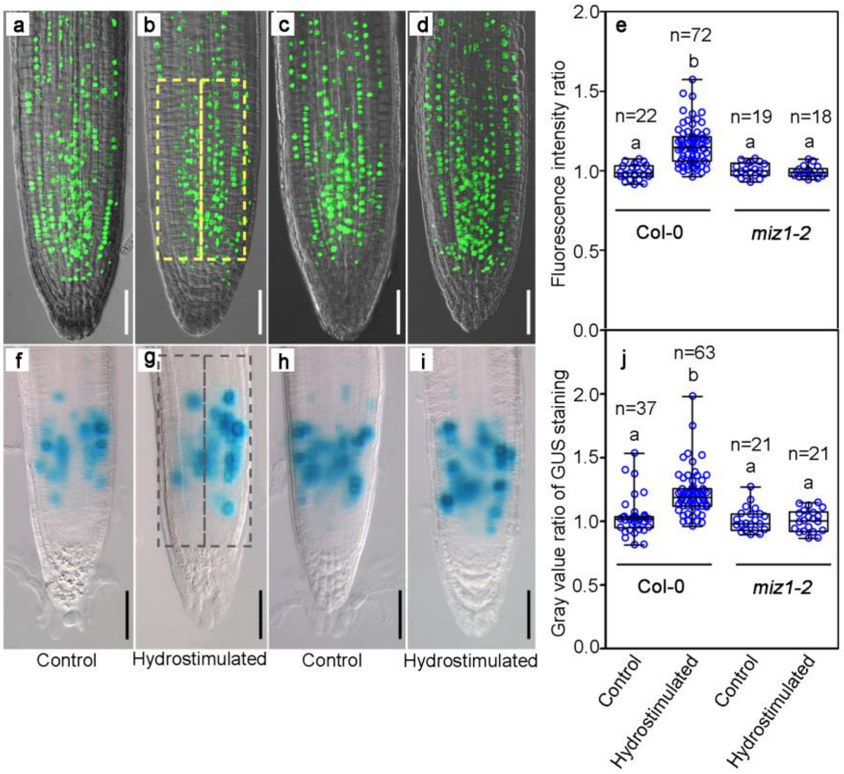

Supplementary information, Fig. S2 Hydrostimulation results in more cell division at convex side than concave side of Col-0 root tips. a-d, DNA replication is analyzed by an EdU staining approach in Col-0 root tips without (**a**) or with (**b**) hydrostimulation. Same approach was also used in *miz1-2* root tips without (**c**) or with (**d**) hydrostimulation treatment. **e**, Measurements of GFP intensity ratio between right and left side (controls) or convex and concave side (hydrostimulated seedlings) within a 50 μm × 200 μm. rectangular area (as shown in **b**) in the root tips above the quiescent center. **f-i**, *pCYCB1;1::GUS* transgenic plants in Col-0 (**f**, **g**) and *miz1-2* (**h**, **i**) backgrounds were used for GUS staining, showing the activities of cell division without (**f**, **h**) or with (**g**, **i**) hydrostimulation. **j**, Measurements of the ratio of GUS gray value between right and left side (controls or *miz1-2*) or convex and concave side (hydrostimulated seedlings) within a 50 μm × 200 μm rectangular area (as shown in **g**) in the root tips above the quiescent center. Each circle represents the measurement from an individual root. Boxplots span the first to the third quartiles of the data. Whiskers indicate minimum and maximum values. A line in the box represents the mean. “n” represents the number of roots used in this experiment. Scale bars represent 50 μm. One-way ANOVA with Tukey’s multiple comparison test was used for statistical analyses. $P < 0.001$.
