# Supplementary material for: Asymmetric distribution of cytokinins determines root hydrotropism in Arabidopsis thaliana
Source: Cell Res. 2019 Oct 10;29(12):984–93. doi: 10.1038/s41422-019-0239-3 (PMC6951336; doi:10.1038/s41422-019-0239-3)
Supplement: Supplementary file 3 — Supplementary information, Figure S3 [file 41422_2019_239_MOESM3_ESM.pdf]

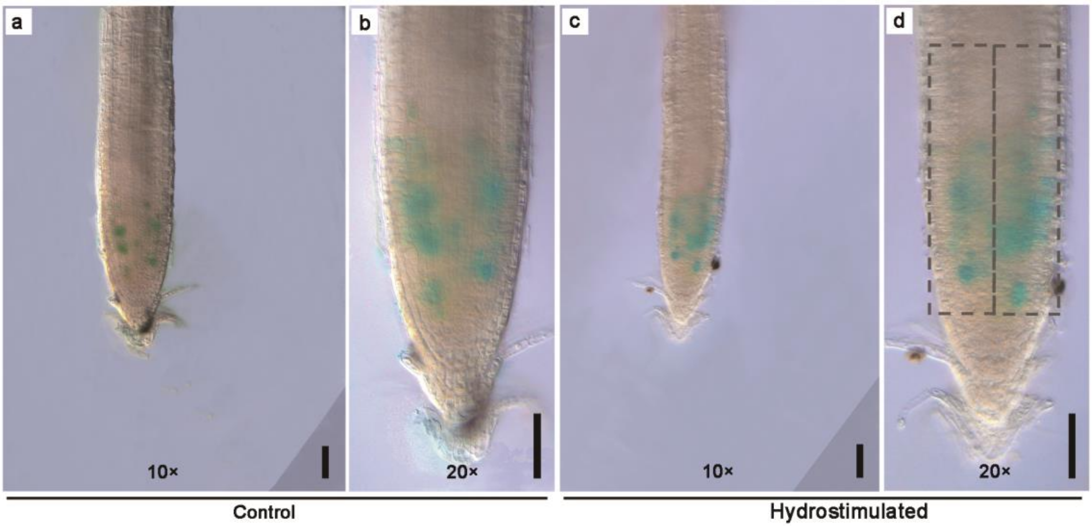

Supplementary information, Fig. S3 On-gel analyses of *pCYCB1;1::GUS* expression without or with hydrostimulation treatment. a-b, both sides of the split agar medium are made by 1/2 MS medium without the supplementation of D-sorbitol. The root in (**b**) is the same root as shown in (**a**) but with different magnification. **c-d**, hydrostimulated roots growing on split agar medium with 1/2 MS medium at the up left side and 1/2 MS medium containing 800 mM D-sorbitol at the bottom right side of the medium. The root in (**d**) is the same root as shown in (**c**) but with different magnification.
