# Supplementary material for: Asymmetric distribution of cytokinins determines root hydrotropism in Arabidopsis thaliana
Source: Cell Res. 2019 Oct 10;29(12):984–93. doi: 10.1038/s41422-019-0239-3 (PMC6951336; doi:10.1038/s41422-019-0239-3)
Supplement: Supplementary file 4 — Supplementary information, Figure S4 [file 41422_2019_239_MOESM4_ESM.pdf]

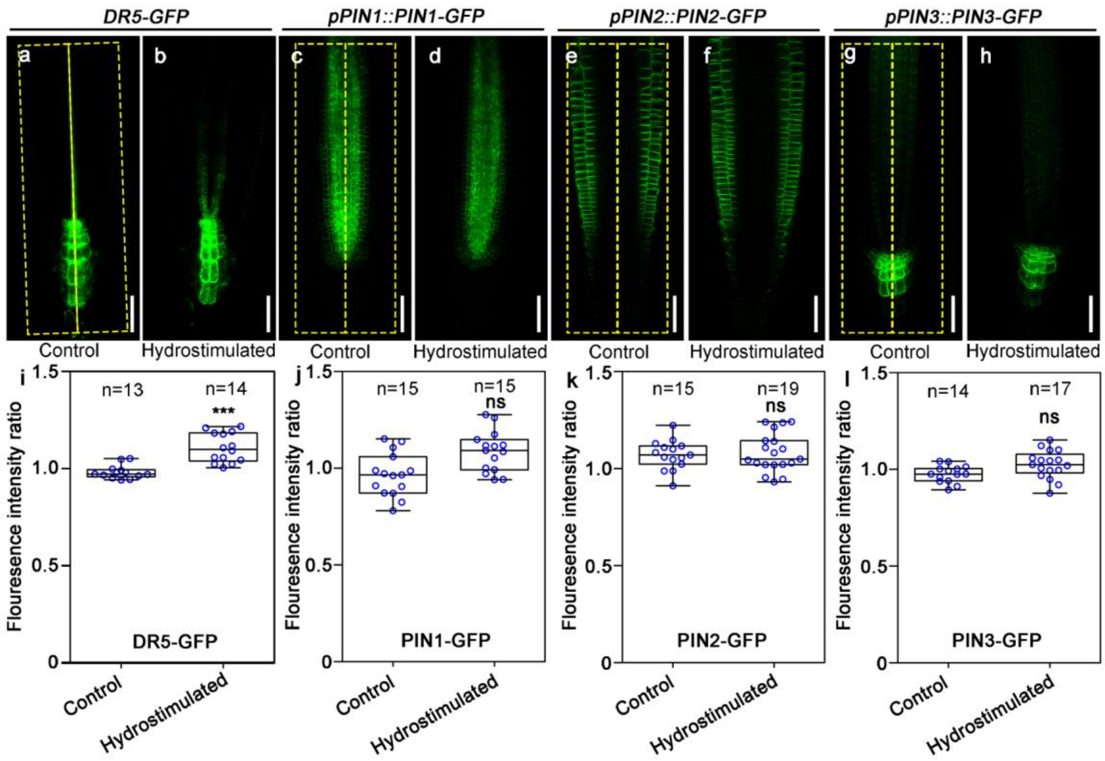

Supplementary information, Fig. S4 Redistribution of auxin and its transporters after hydrostimulation treatment. a-b, DR5-GFP redistribution after hydrostimulation with (**a**) as a control and (**b**) as a hydrostimulated root. **c-d**, PIN1-GFP redistribution after hydrostimulation with (**c**) as a control and (**d**) as a hydrostimulated root. **e-f**, PIN2-GFP redistribution after hydrostimulation with (**e**) as a control and (**f**) as a hydrostimulated root. **g-h**, PIN3-GFP redistribution after hydrostimulation with (**g**) as a control and (**h**) as a hydrostimulated root. **i-l**, measurements of fluorescence intensity ratio (right/left for controls or convex/concave for hydrostimulated roots) for DR5-GFP (**i**), PIN1-GFP (**j**), PIN2-GFP (**k**), and PIN3-GFP (**l**). The fluorescence was measured in a 60 µm × 400 µm area as shown in (**a-h**). Each circle represents the measurement from an individual root. Boxplots span the first to third quartiles of the data. Whiskers indicate minimum and maximum values. A line in the box represents the mean. “n” represents the number of roots used in this experiment. Scale bars represent 50 µm. Student’s t test, was used for statistical analyses. ‘ns’ represents no significant difference. $P < 0.0001$.
