# Supplementary material for: Asymmetric distribution of cytokinins determines root hydrotropism in Arabidopsis thaliana
Source: Cell Res. 2019 Oct 10;29(12):984–93. doi: 10.1038/s41422-019-0239-3 (PMC6951336; doi:10.1038/s41422-019-0239-3)
Supplement: Supplementary file 5 — Supplementary information, Figure S5 [file 41422_2019_239_MOESM5_ESM.pdf]

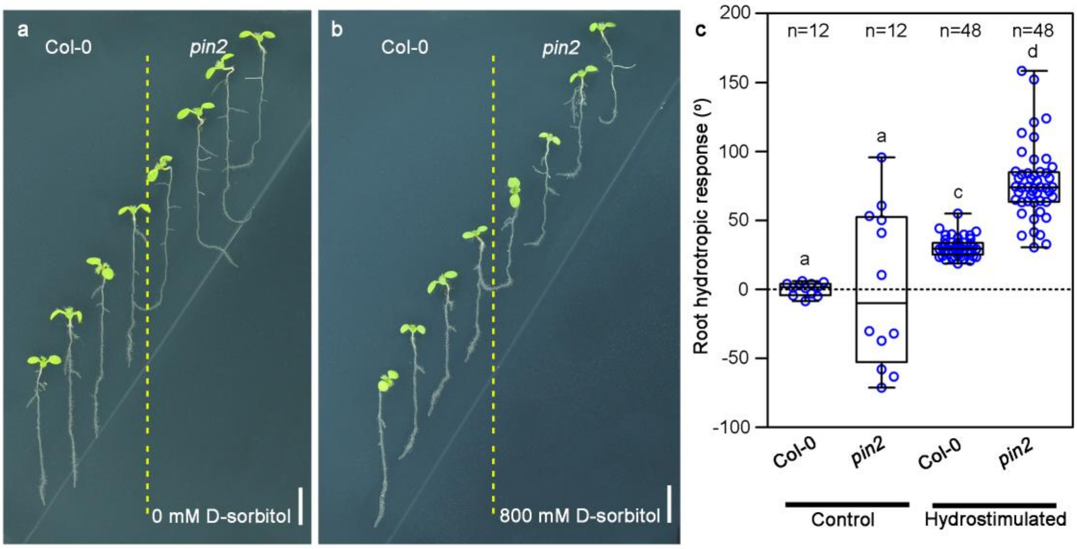

Supplementary information, Fig. S5 The polar transportation of auxin is not required for root hydrotropism. a, Col-0 and *pin2* roots growing on split agar medium with both sides made by 1/2 MS medium without the supplementation of D-sorbitol. **b**, Col-0 and *pin2* roots growing on split agar medium with 1/2 MS medium at the up left side and 1/2 MS medium containing 800 mM D-sorbitol at the bottom right side of the medium. **c**, Measurements of the root growth orientation in response to hydrostimulation treatment. Each circle represents the measurement from an individual root. Boxplots span the first to third quartiles of the data. Whiskers indicate minimum and maximum values. A line in the box represents the mean. “n” represents the number of roots used in this experiment. Scale bars represent 50 µm. One-way ANOVA with Tukey’s multiple comparison test was used for statistical analyses. $P < 0.001$.
