# Supplementary material for: Asymmetric distribution of cytokinins determines root hydrotropism in Arabidopsis thaliana
Source: Cell Res. 2019 Oct 10;29(12):984–93. doi: 10.1038/s41422-019-0239-3 (PMC6951336; doi:10.1038/s41422-019-0239-3)
Supplement: Supplementary file 6 — Supplementary information, Figure S6 [file 41422_2019_239_MOESM6_ESM.pdf]

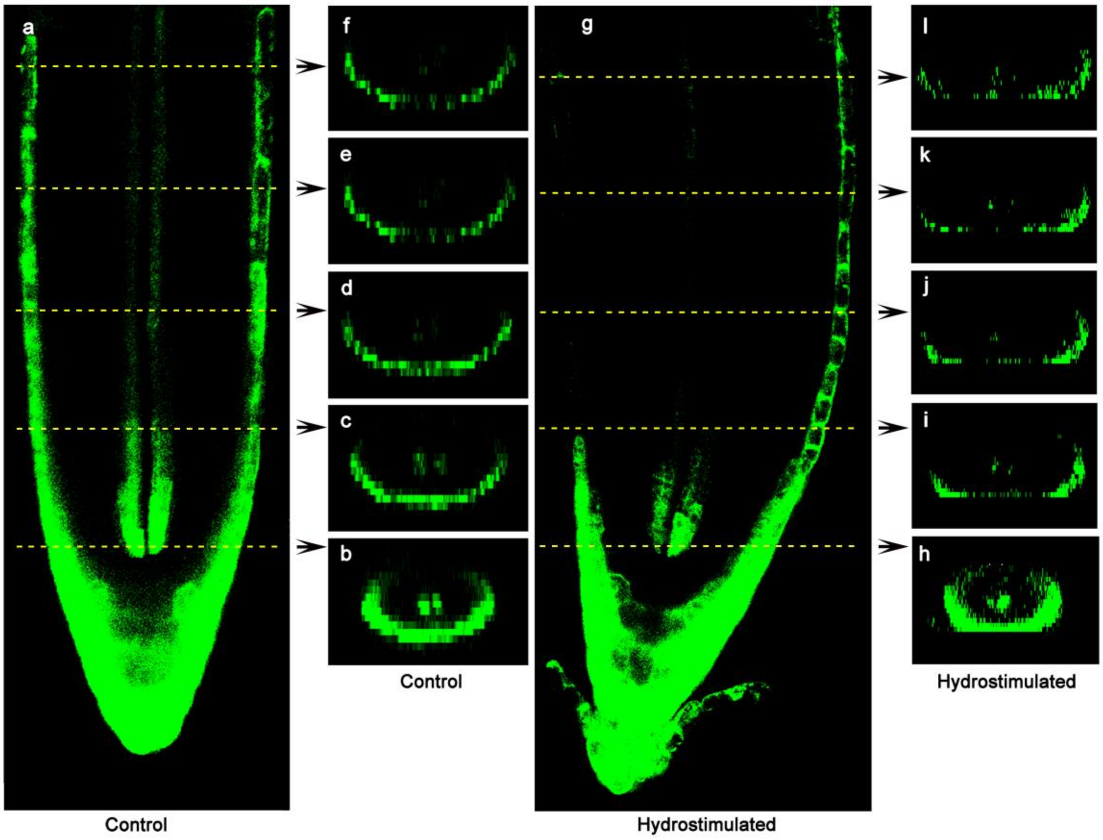

Supplementary information, Fig. S6 More cytokinin response was observed at the lower water potential side of the Col-0 root tip after hydrostimulation. a, *TCSn::GFP* is symmetrically expressed in longitudinally viewed root tips in control seedlings. **b-f**, Transverse views of the root GFP signals showed in **a**, the section positions are 0 µm (**b**), 50 µm (**c**), 100 µm (**d**), 150 µm (**e**), 200 µm (**f**) from the quiescent center. **g**, Asymmetric expression of *TCSn::GFP* in longitudinally viewed root tips after hydrostimulation. **h-l**, Transverse views of the root showed in **g**, the section positions are 0 µm (**h**), 50 µm (**i**), 100 µm (**j**), 150 µm (**k**), 200 µm (**l**) from the quiescent center.
