# Supplementary material for: Asymmetric distribution of cytokinins determines root hydrotropism in Arabidopsis thaliana
Source: Cell Res. 2019 Oct 10;29(12):984–93. doi: 10.1038/s41422-019-0239-3 (PMC6951336; doi:10.1038/s41422-019-0239-3)
Supplement: Supplementary file 7 — Supplementary information, Figure S7 [file 41422_2019_239_MOESM7_ESM.pdf]

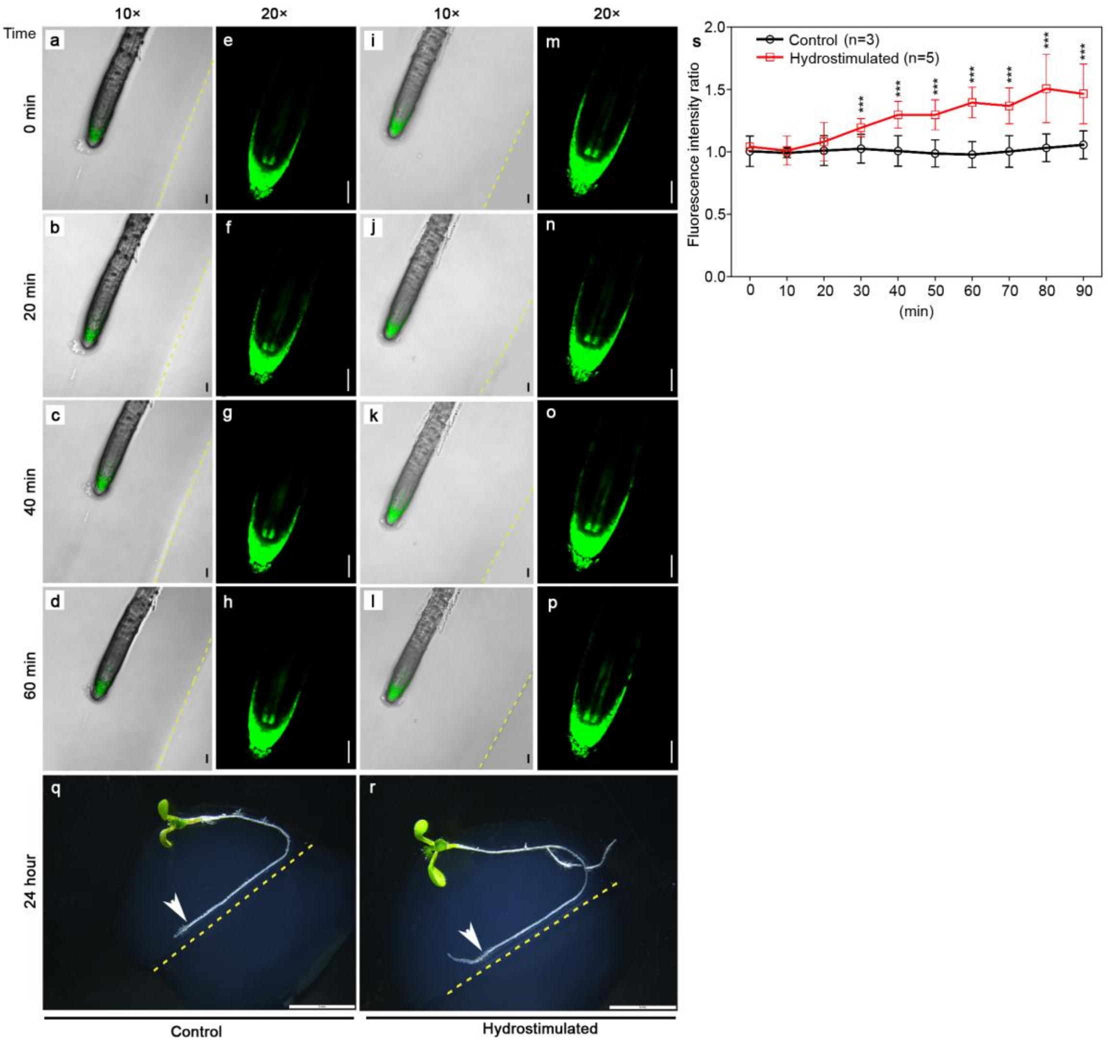

Supplementary information, Fig. S7 Representative on-gel analyses of *TCSn::GFP* expression during hydrostimulation treatment. Four-day-old seedlings were moved to a split agar medium (1/2 MS-1/2MS for control, 1/2 MS-1/2 MS containing 800 mM D-sorbitol for hydrostimulation treatment) and the root tip GFP signal was photographed within a 60-min period with a 20-min interval. **a**-**h**, GFP signal of a representative root growing on control split agar medium. All the images are from the same root with different magnifications. **i**-**p**, GFP signal of a representative root growing on hydrostimulation split agar medium. All the images are from the same root with different magnifications. **q**, Root tip growth orientation of the same root as shown in (**a**-**h**)after 24 hours. **r**, root tip growth orientation of the same root as shown in (**i**-**p**) after 24 h hydrostimulation treatment. The dotted line in figures indicates the medium bend of split-agar medium. Scale bars represent 50 μm in (**a**-**p**) and 5 mm in (**q**-**r**). **s**, Measurements of fluorescence intensity ratio (right/left for controls or lower water potential/ higher water potential for hydrostimulated roots) within a 90-min period with a 10-min interval. The fluorescence intensity was measured within a 200 μm meristematic zone starting from the quiescent center. Student's *t* test was used for statistical analyses. "n" represents the number of roots used in this experiment. $P < 0.01$.
