# Supplementary material for: Asymmetric distribution of cytokinins determines root hydrotropism in Arabidopsis thaliana
Source: Cell Res. 2019 Oct 10;29(12):984–93. doi: 10.1038/s41422-019-0239-3 (PMC6951336; doi:10.1038/s41422-019-0239-3)
Supplement: Supplementary file 8 — Supplementary information, Figure S8 [file 41422_2019_239_MOESM8_ESM.pdf]

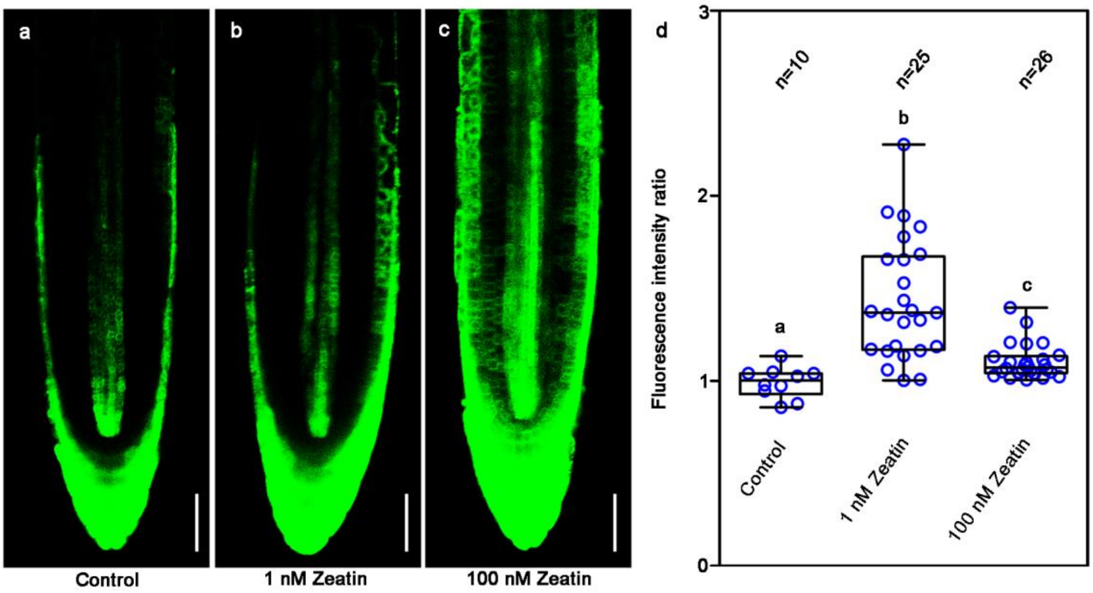

Supplementary information, Fig. S8 One side zeatin treatment on a spilt-agar medium can effectively induce asymmetric *TCSn::GFP* response in root tips. a-c, Root tip images showing GFP signals after one side zeatin treatment with 0 nM, 1 nM, and 100 nM zeatin at the bottom right side of the split-agar medium of one hour. **d**, The measurements of fluorescence intensity ratio (right/left for control and zeatin/ non-zeatin side for one-side zeatin treatments). Each circle represents the measurement from an individual root. Boxplots span the first to third quartiles of the data. Whiskers indicate minimum and maximum values. A line in the box represents the mean. “n” represents the number of roots used in this experiment. Scale bars represent 50 µm. One-way ANOVA with Tukey’s multiple comparison test was used for statistical analyses. $P < 0.001$.
