# Supplementary material for: Asymmetric distribution of cytokinins determines root hydrotropism in Arabidopsis thaliana
Source: Cell Res. 2019 Oct 10;29(12):984–93. doi: 10.1038/s41422-019-0239-3 (PMC6951336; doi:10.1038/s41422-019-0239-3)
Supplement: Supplementary file 9 — Supplementary information, Figure S9 [file 41422_2019_239_MOESM9_ESM.pdf]

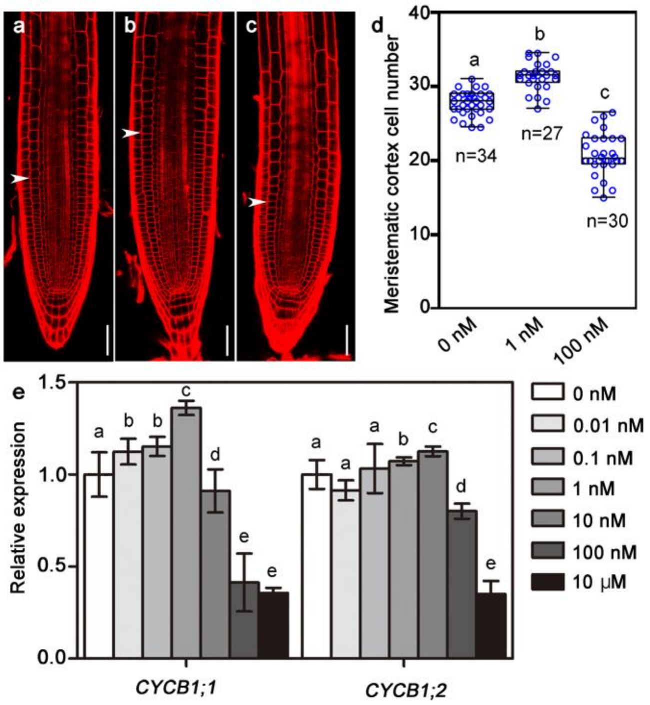

Supplementary information, Fig. S9 Low concentration of cytokinins is able to stimulate cell division in meristematic zone and high concentration cytokinin can inhibit it. a-c, Representative propidium iodide-stained four-day-old Col-0 root tips treated with the 1/2 MS medium containing 0 nM (**a**), 1 nM (**b**), or 100 nM (**c**) zeatin for one day. White arrow heads mark the junction of meristem and elongation zones. **d**, Measurements of meristematic cortex cell numbers after zeatin treatments as shown in (**a-c**). Each circle represents the measurement from an individual root. Boxplots span the first to third quartiles of the data. Whiskers indicate minimum and maximum values. A line in the box represents the mean. “n” represents the number of roots used in this experiment. One-way ANOVA with Tukey’s multiple comparison test was used for statistical analyses. $P < 0.001$. **e**, Real-time RT-PCR analyses showing the expression of *CYCB1;1* and *CYCB1;2*, marker genes for cell division, in response to different concentrations of zeatin treatments. Four-day-old seedlings were transferred from 1/2 MS supplemented with 1% agar and 1% sucrose to the same 1/2 MS medium with additional different concentrations of zeatin. After 16 hours, 0.5 cm root tips were collected for total RNA extraction. The total RNAs were used for qRT-PCR analyses. Scale bars represent 50 µm. Gene expression levels were shown as mean ± SD (n = 3). *ACTIN2* was used as an internal control. One-way ANOVA with Tukey’s multiple comparison test was used for statistical analyses. $P < 0.001$.
