# Supplementary material for: Asymmetric distribution of cytokinins determines root hydrotropism in Arabidopsis thaliana
Source: Cell Res. 2019 Oct 10;29(12):984–93. doi: 10.1038/s41422-019-0239-3 (PMC6951336; doi:10.1038/s41422-019-0239-3)
Supplement: Supplementary file 10 — Supplementary information, Figure S10 [file 41422_2019_239_MOESM10_ESM.pdf]

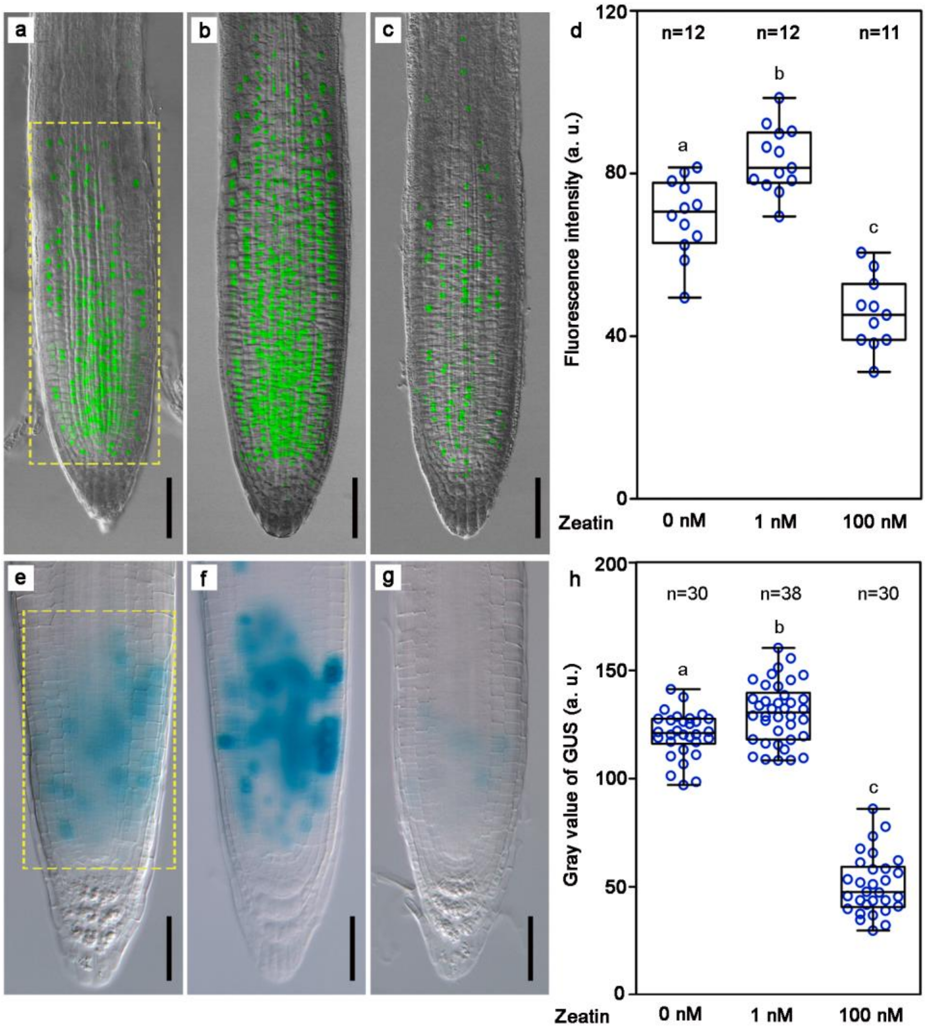

Supplementary information, Fig. S10 Low concentration of cytokinins can stimulate cell division in root meristematic zone and high concentration cytokinin can inhibit it. a-c, DNA replication is analyzed by an EdU staining approach in Col-0 root tips treated with 0 nM (**a**), 1 nM (**b**), or 100 nM zeatin (**c**). **d**, Measurements of GFP intensity of EdU staining within a 120 µm × 300 µm rectangular area (as shown in **a**) in the root tips above the quiescent center. **e-g**, GUS staining of the *pCYCB1;1::GUS* transgenic root tips treated with 0 nM (**e**), 1 nM (**f**), or 100 nM zeatin (**g**). **h**, Measurements of GUS gray value within a 120 µm × 200 µm rectangular area (as shown in **e**) in the root tips above the quiescent center. Each circle represents the measurement from an individual root. Boxplots span the first to third quartiles of the data. Whiskers indicate minimum and maximum values. A line in the box represents the mean. “n” represents the number of roots used in this experiment. Scale bars represent 50 µm. One-way ANOVA with Tukey’s multiple comparison test was used for statistical analyses. $P < 0.001$.
