# Supplementary material for: Asymmetric distribution of cytokinins determines root hydrotropism in Arabidopsis thaliana
Source: Cell Res. 2019 Oct 10;29(12):984–93. doi: 10.1038/s41422-019-0239-3 (PMC6951336; doi:10.1038/s41422-019-0239-3)
Supplement: Supplementary file 11 — Supplementary information, Figure S11 [file 41422_2019_239_MOESM11_ESM.pdf]

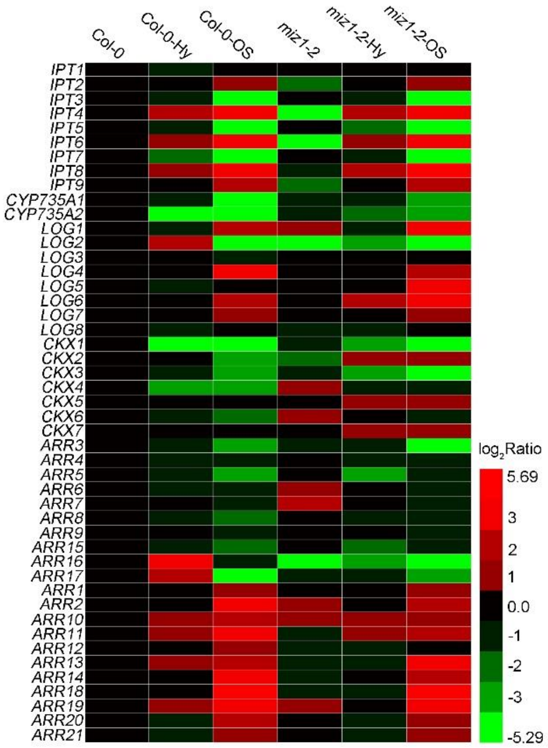

Supplementary information, Fig. S11 qRT-PCR results showing the transcriptions of genes encoding cytokinin biosynthesis, metabolism, type-A, and type-B response regulators without or with hydrostimulation treatment. a-c, Real-time RT-PCR analyses showing the expression of cytokinin biosynthetic genes, such as *IPT*s, *CYP735A*s, and *LOG*s, cytokinin metabolic genes, *CKX*s, and type-A and type-B response regulators in response to hydrostimulation treatment (Hy) and osmotic stress treatment (OS) in Col-0 and *miz1-2* root tips. plants treated with medium containing 800 Mm D-sorbitol was defined as 'osmotic stress' treatment. Three biological replicates were carried out. The data shown are from one representative experiment. Each analysis contains three technical repeats.
