# Supplementary material for: Asymmetric distribution of cytokinins determines root hydrotropism in Arabidopsis thaliana
Source: Cell Res. 2019 Oct 10;29(12):984–93. doi: 10.1038/s41422-019-0239-3 (PMC6951336; doi:10.1038/s41422-019-0239-3)
Supplement: Supplementary file 12 — Supplementary information, Figure S12 [file 41422_2019_239_MOESM12_ESM.pdf]

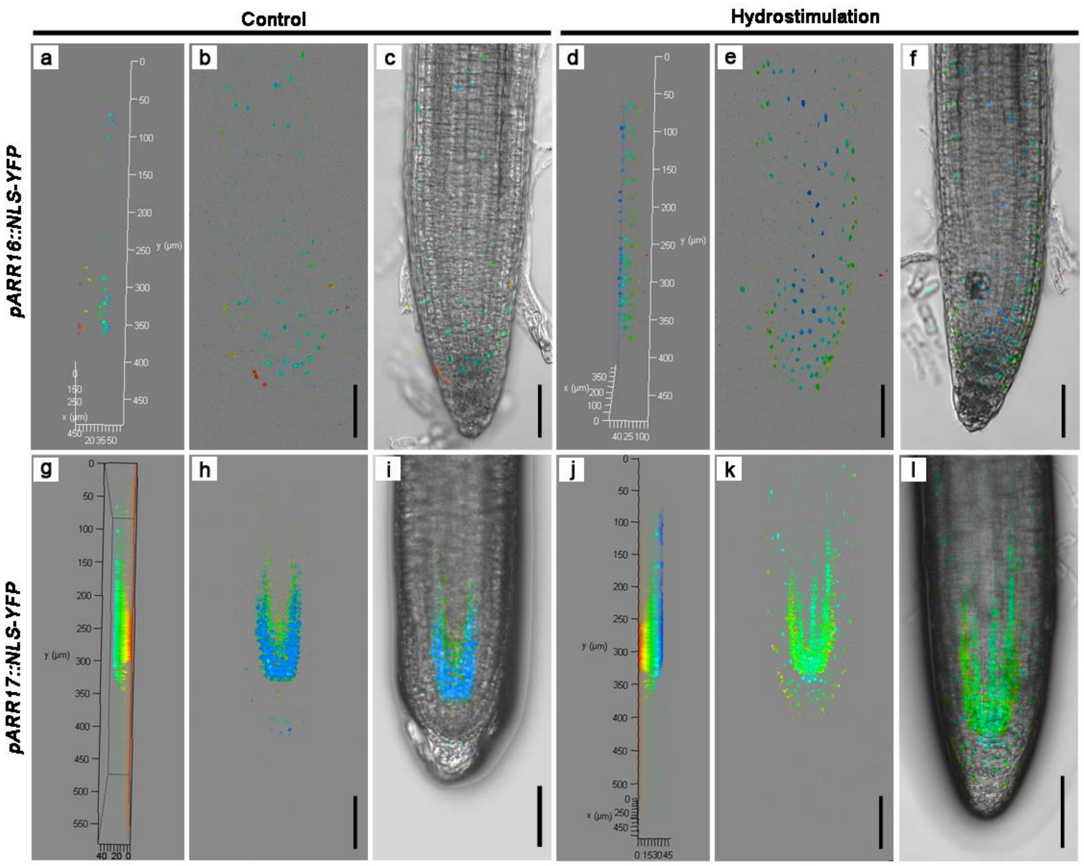

Supplementary information, Fig. S12 Stacked multilayer scanning of root tips from *pARR16*::NLS-YFP and *pARR17::NLS-YFP* transgenic seedlings under on-gel hydrostimulation treatment. a-f, Four-day-old *pARR16::NLS-YFP* transgenic seedlings were moved to a split agar medium (1/2 MS-1/2MS for control, 1/2 MS-1/2 MS containing 800 mM D-sorbitol for hydrostimulation treatment) and the root tip NLS-YFP signal was scanned layer-by-layer. The depth of the scanning is shown in (**a**, **d**)**.** Different color represents the signal from different layer. The stacked signal from all different layers is shown in (**b**, **e**). The merged stacked NLS-YFP signals with the bright-field root image is shown in (**c**, **f**). **g-k**, Four-day-old *pARR17::NLS-YFP* transgenic seedlings were moved to a split agar medium (1/2 MS-1/2MS for control, 1/2 MS-1/2 MS containing 800 mM D-sorbitol for hydrostimulation treatment) and the root tip NLS-YFP signal was scanned layer-by-layer. The depth of the scanning is shown in (**g**, **j**)**.** Different color represents the signal from different layer. The stacked signal from all different layers is shown in (**h**, **k**). The merged image showing stacked NLS-YFP signals with the bright-field root image is shown in (**i**, **l**).
