# Supplementary material for: Asymmetric distribution of cytokinins determines root hydrotropism in Arabidopsis thaliana
Source: Cell Res. 2019 Oct 10;29(12):984–93. doi: 10.1038/s41422-019-0239-3 (PMC6951336; doi:10.1038/s41422-019-0239-3)
Supplement: Supplementary file 14 — Supplementary information, Figure S14 [file 41422_2019_239_MOESM14_ESM.pdf]

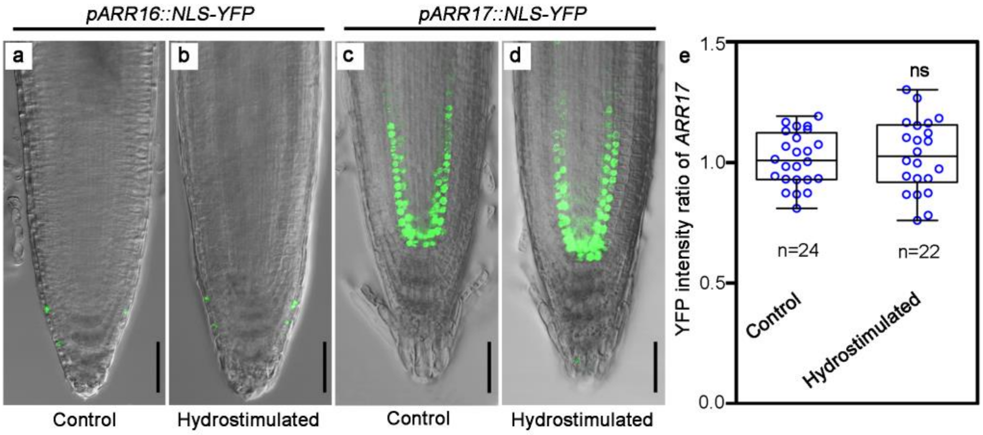

Supplementary information, Fig. S14 The expression of *ARR16* and *ARR17* showed no significant responses to hydrostimulation treatment in *miz1-2*. a-d, Confocal analyses showing the expression of *pARR16::NLS-YFP* (**a**, **b**), and *pARR17::NLS-YFP* (**c**, **d**) in their corresponding transgenic plants in *miz1-2* background after hydrostimulation treatment (**b**, **d**). **e**, Ratio of YFP intensity in *pARR17::NLS-YFP* between right side and left side in the endodermis cell layer within a 200-μm meristematic zone starting from the quiescent center. Each circle represents the measurement from an individual root. Boxplots span the first to third quartiles of the data. Whiskers indicate minimum and maximum values. A line in the box represents the mean. “n” represents the number of roots used in this experiment. Scale bars represent 50 μm. Student’s *t* test was used for statistical analyses. ‘ns’ represents no significant difference. $P < 0.0001$
