# Supplementary material for: Asymmetric distribution of cytokinins determines root hydrotropism in Arabidopsis thaliana
Source: Cell Res. 2019 Oct 10;29(12):984–93. doi: 10.1038/s41422-019-0239-3 (PMC6951336; doi:10.1038/s41422-019-0239-3)
Supplement: Supplementary file 15 — Supplementary information, Figure S15 [file 41422_2019_239_MOESM15_ESM.pdf]

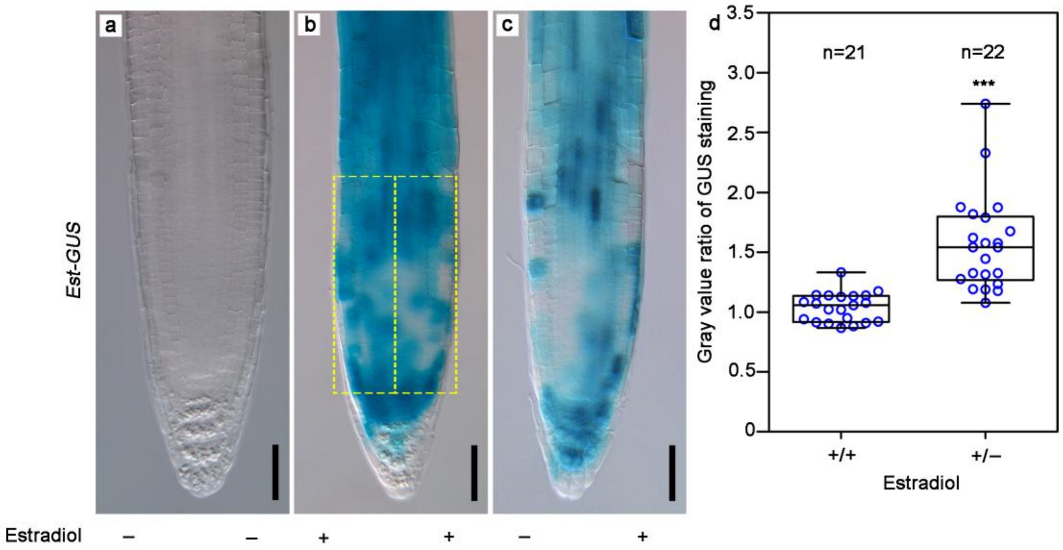

Supplementary information, Fig. S15 An asymmetric inducible system is sufficient to induce asymmetric expression of target genes. a-c, GUS staining of the roots from *Est-GUS* transgenic seedlings without (**a**), with estradiol on both sides (**b**), or with estradiol at the bottom right side of the split-agar medium (**c**). **d,** Measurements of the GUS signal ratio (right/left) after both sides or one-side of the roots were treated with 50 µM estradiol in an area of 200 µm × 60 µm in the root meristem zone (as shown in **b**). Each circle represents the measurement from an individual root. Boxplots span the first to third quartiles of the data. Whiskers indicate minimum and maximum values. A line in the box represents the mean. “n” represents the number of roots used in this experiment. Scale bars represent 50 µm. One-way ANOVA with Tukey’s multiple comparison test was used for statistical analyses. $P < 0.001$.
