# Supplementary material for: Asymmetric distribution of cytokinins determines root hydrotropism in Arabidopsis thaliana
Source: Cell Res. 2019 Oct 10;29(12):984–93. doi: 10.1038/s41422-019-0239-3 (PMC6951336; doi:10.1038/s41422-019-0239-3)
Supplement: Supplementary file 16 — Supplementary information, Figure S16 [file 41422_2019_239_MOESM16_ESM.pdf]

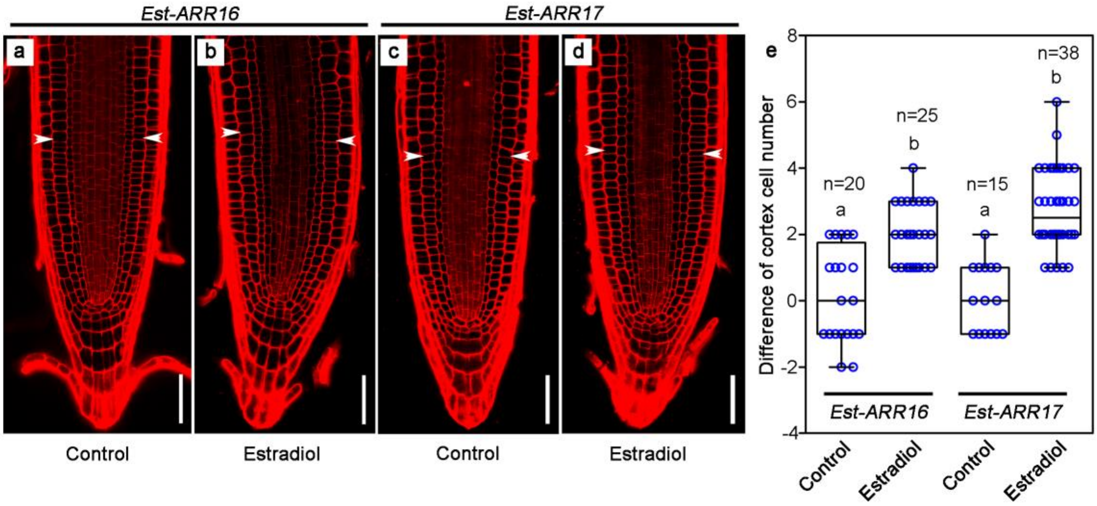

Supplementary information, Fig. S16 Induced asymmetric expression of *ARR16* or *ARR17* results in more cell division at the induced side of the roots. a, Representative propidium iodide-stained root of four-day-old *Est-ARR16* transgenic plant in Col-0 background after treated with DMSO at the bottom right side of the medium for 2 hours. **b**, Representative propidium iodide-stained root of four-day-old *Est-ARR16* transgenic plant in Col-0 background after treated with 50 μM estradiol at the bottom right side of the medium for 2 hours. **c**, Representative propidium iodide-stained root of four-day-old *Est-ARR17* transgenic plant in Col-0 background after treated with DMSO at the bottom right side for 2 hours. **d**, Representative propidium iodide-stained root of four-day-old *Est-ARR17* transgenic plant in Col-0 background after treated with 50 μM estradiol at the bottom right side of the medium for 2 hours. **e**, Measurements of cortex cell number differences between right side and left side (control) or estradiol-treated side and untreated side (estradiol treatment) within a 200-μm meristematic region (as marked by white arrow heads) starting from the quiescent center. Each circle represents the measurement from an individual root. Boxplots span the first to third quartiles of the data. Whiskers indicate minimum and maximum values. A line in the box represents the mean. “n” represents the number of roots used in this experiment. Scale bars represent 50 μm. One-way ANOVA with Tukey’s multiple comparison test was used for statistical analyses. $P < 0.001$.
