# Supplementary material for: Asymmetric distribution of cytokinins determines root hydrotropism in Arabidopsis thaliana
Source: Cell Res. 2019 Oct 10;29(12):984–93. doi: 10.1038/s41422-019-0239-3 (PMC6951336; doi:10.1038/s41422-019-0239-3)
Supplement: Supplementary file 17 — Supplementary information, Figure S17 [file 41422_2019_239_MOESM17_ESM.pdf]

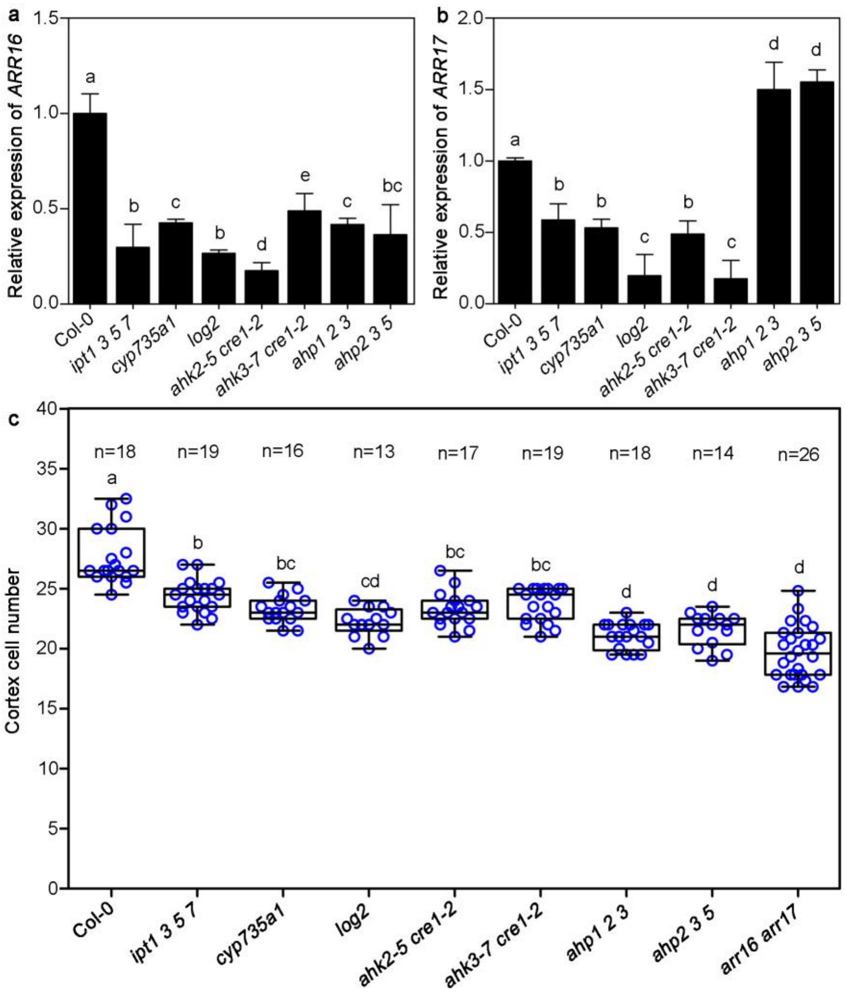

Supplementary information, Fig. S17 Expression levels of *ARR16* and *ARR17* and cell division are significantly decreased in the root tips of cytokinin biosynthesis and signaling mutants. a, Relative expression levels of *ARR16* in root tips of wild-type and various cytokinin biosynthesis and signaling mutant seedlings. **b**, Relative expression levels of *ARR17* in root tips of wild-type and various cytokinin biosynthesis and signaling mutant seedlings. **c**, cortex cell numbers within 200 µm distance above the quiescent center in wild-type and various cytokinin biosynthesis and signaling mutants. Each circle represents the measurement from an individual root. Boxplots span the first to third quartiles of the data. Whiskers indicate minimum and maximum values. A line in the box represents the mean. "n" represents the number of roots used in this experiment. One-way ANOVA with Tukey's multiple comparison test was used for statistical analyses. $P < 0.001$.
