# Supplementary material for: Asymmetric distribution of cytokinins determines root hydrotropism in Arabidopsis thaliana
Source: Cell Res. 2019 Oct 10;29(12):984–93. doi: 10.1038/s41422-019-0239-3 (PMC6951336; doi:10.1038/s41422-019-0239-3)
Supplement: Supplementary file 18 — Supplementary information, Figure S18 [file 41422_2019_239_MOESM18_ESM.pdf]

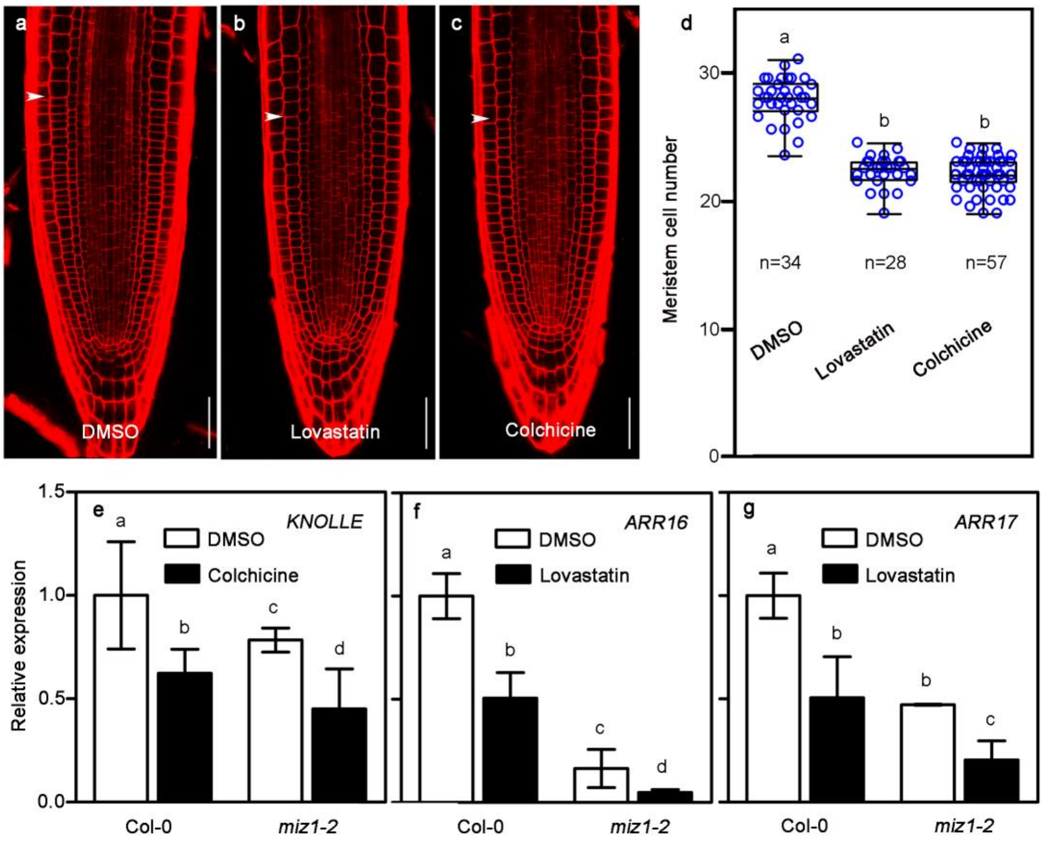

Supplementary information, Fig. S18 Treatment of either lovastatin or colchicine can effectively inhibit root cell division. a-c, Representative propidium iodide stained four-day-old Col-0 root tips grown on 1/2 MS medium containing DMSO (**a**), lovastatin (**b**), and colchicine (**c**) for one day. White arrow heads mark the junction of meristem and elongation zones **d**, Measurements of meristematic cortex cell numbers after treatments as shown in (**a-c**). Each circle represents the measurement from an individual root. Boxplots span the first to third quartiles of the data. Whiskers indicate minimum and maximum values. A line in the box represents the mean. "n" represents the number of roots used in this experiment. **e-g**, qRT-PCR analyses showing the expression of a gene specifically expressed in plant dividing cells, *KNOLLE*[1], in response to DMSO or colchicine(**e**) and two cytokinin response regulators *ARR16* (**f**) and *ARR17* (**g**) in response to DMSO or lovastatin treatment in Col-0 and *miz1-2* root tips. Gene expression was shown as mean ± SD (n = 3). *ACTIN2* was analyzed as an internal control. Scale bars represent 50 µm. One-way ANOVA with Tukey's multiple comparison test was used for statistical analyses. $P < 0.001$.

SUPPLEMENTARY REFERENCE

1. Touihri, S. *et al.* Functional anatomy of the Arabidopsis cytokinesis-specific syntaxin KNOLLE. *Plant J* **68**, 755-764, (2011).
