# Supplementary material for: Asymmetric distribution of cytokinins determines root hydrotropism in Arabidopsis thaliana
Source: Cell Res. 2019 Oct 10;29(12):984–93. doi: 10.1038/s41422-019-0239-3 (PMC6951336; doi:10.1038/s41422-019-0239-3)
Supplement: Supplementary file 19 — Supplementary information, Figure S19 [file 41422_2019_239_MOESM19_ESM.pdf]

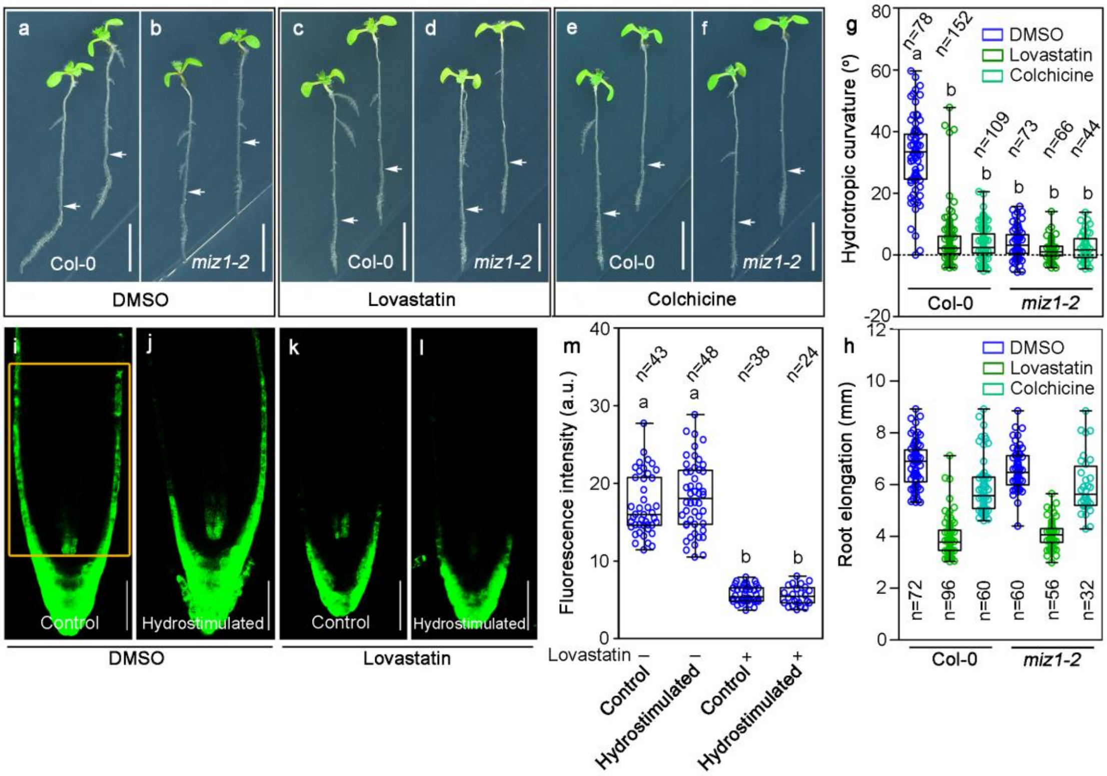

Supplementary information, Fig. S19 Biosynthesis of cytokinins and cell division are essential for root hydrotropic response. a, **c**, **e**, Representative root growth curvatures after three-day-old Col-0 seedlings were pretreated on 1/2 MS medium with DMSO, lovastatin, or colchicine for one day, and then placed on hydrostimulating medium containing DMSO (**a**), lovastatin (**c**), and colchicine (**e**) for additional one (DMSO) or two days (lovastatin or colchicine). **b**, **d**, **f**, Root growth curvatures of *miz1-2* after the same treatments as Col-0. **g**, Measurements of root growth curvatures of Col-0 and *miz1-2* seedlings upon the treatments as shown in **a**-**f**. White arrows in **a**-**f** indicate the root tip positions right after transferring the seedlings to the hydrostimulating medium. **h**, Measurements of root elongation after one day (DMSO) or two days (lovastatin or colchicine) of hydrostimulation treatment. **i**-**l**, Confocal data indicating lovastatin treatment can effectively inhibit the biosynthesis of cytokinins, as visualized by the GFP signal using a representative *TCSn::GFP* transgenic line in Col-0. Three-day-old seedlings were transferred to 1/2 MS medium containing DMSO (**i**, **j**) or lovastatin (**k**, **l**), pretreated for one day, and then transferred to either control medium (**i**, **k**) or hydrostimulating medium (**j**, **l**), without (**j**) or with (**l**) lovastatin, for an additional 1 hour before performing confocal analyses. **m**, Measurements of fluorescence intensity using a Leica confocal software in an area of 200 μm × 120 μm in the root meristem zone (as shown in **i**). Each circle represents the measurement from an individual root. Boxplots span the first to third quartiles of the data. Whiskers represent minimum and maximum values. A line in the box represents the mean. “n” represents the number of roots used in this experiment. Scale bars represent 5 mm in (**a**-**f**), and 50 μm in (**i**-**l**). One-way ANOVA with Tukey’s multiple comparison test was used for statistical analyses. $P<0.001$.
