# Supplementary material for: Asymmetric distribution of cytokinins determines root hydrotropism in Arabidopsis thaliana
Source: Cell Res. 2019 Oct 10;29(12):984–93. doi: 10.1038/s41422-019-0239-3 (PMC6951336; doi:10.1038/s41422-019-0239-3)
Supplement: Supplementary file 20 — Supplementary information, Figure S20 [file 41422_2019_239_MOESM20_ESM.pdf]

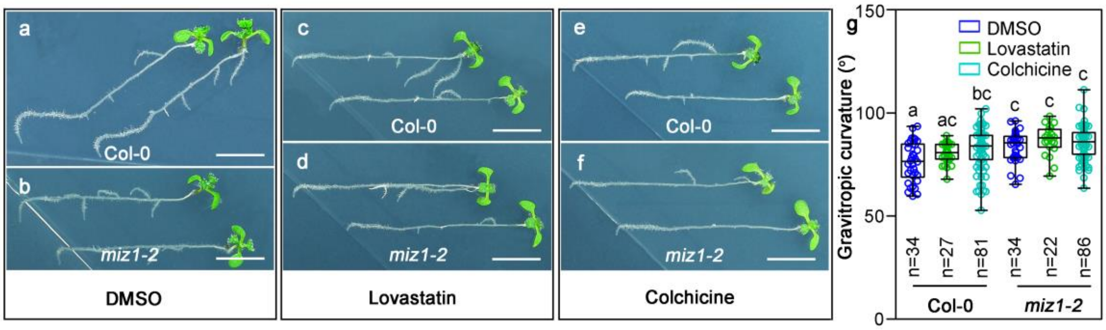

Supplementary information, Fig. S20 Inhibition of either the biosynthesis of cytokinins or cell division does not affect root gravitropic response. a-**f**, Root gravitropism of Col-0 (**a**, **c**, **e**) and *miz1-2* (**b**, **d**, **f**). Petri dishes after hydrostimulation experiments explained in Supplementary information Fig. S19 were rotated clockwise to make root tips horizontally placed and the petri dishes were vertically incubated for an additional one day to analyze their gravitropic responses. **g**, Measurements of root gravitropism of Col-0 and *miz1-2* upon the treatments as shown in (**a**-**f**). Each circle represents the measurement from an individual root. Boxplots span the first to third quartiles of the data. Whiskers indicate minimum and maximum values. A line in the box represents the mean. “n” represents the number of roots used in this experiment. Scale bars represent 5 mm. One-way ANOVA with Tukey’s multiple comparison test was used for statistical analyses. $P < 0.001$.
