# Supplementary material for: Asymmetric distribution of cytokinins determines root hydrotropism in Arabidopsis thaliana
Source: Cell Res. 2019 Oct 10;29(12):984–93. doi: 10.1038/s41422-019-0239-3 (PMC6951336; doi:10.1038/s41422-019-0239-3)
Supplement: Supplementary file 21 — Supplementary information, Figure S21 [file 41422_2019_239_MOESM21_ESM.pdf]

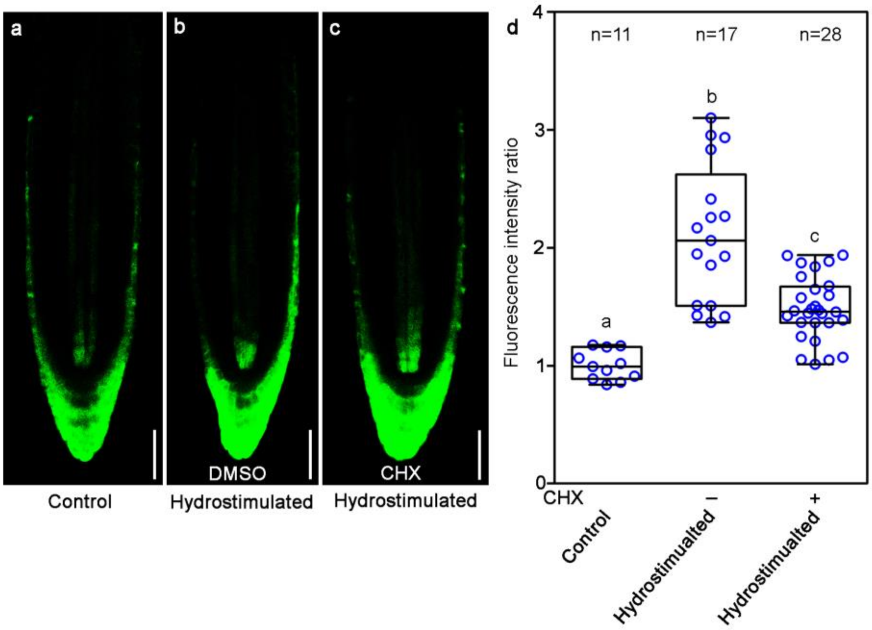

Supplementary information, Fig. S21 Asymmetric distribution or response of cytokinins partially relies on the synthesis of new proteins. a-c, Asymmetric distribution of cytokinin response (GFP signal) under different treatments including the control (**a**), hydrostimulation supplemented with DMSO (**b**) or supplemented with 20 µM CHX in DMSO (**c**) for 60 min. **d**, GFP fluorescence ratio either between right side and left side of lateral root caps (controls), or between lower water potential side and higher water potential side of lateral root caps (hydrostimulated, with or without the treatment of CHX) within a 200-µm meristematic zone starting from the quiescent center. Each circle represents the measurement from an individual root. Boxplots span the first to the third quartiles of the data. Whiskers indicate minimum and maximum values. A line in the box represents the mean. “n” represents the number of roots used in this experiment. Scale bars represent 50 µm. One-way ANOVA with Tukey’s multiple comparison test was used for statistical analyses. $P < 0.001$.
