# Supplementary material for: Asymmetric distribution of cytokinins determines root hydrotropism in Arabidopsis thaliana
Source: Cell Res. 2019 Oct 10;29(12):984–93. doi: 10.1038/s41422-019-0239-3 (PMC6951336; doi:10.1038/s41422-019-0239-3)
Supplement: Supplementary file 22 — Supplementary information, Figure S22 [file 41422_2019_239_MOESM22_ESM.pdf]

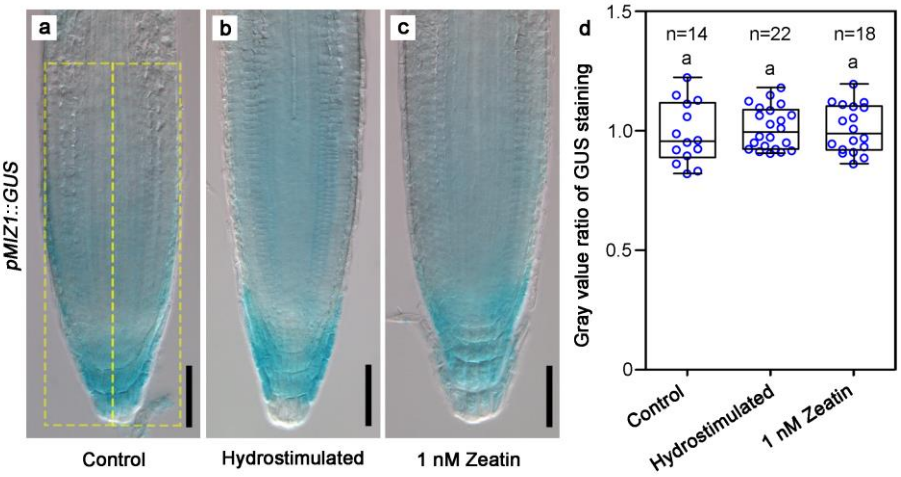

Supplementary information, Fig. S22 The expression of *MIZ1* showed no asymmetric distribution after hydrostimulation or one-side cytokinin treatment. **a**-**c**, GUS staining after *pMIZ1::GUS* seedlings were untreated (**a**) or treated with hydrostimulation (**b**) or one-side 1 nM zeatin (**c**) in split-agar media. **d,** Measurements of the GUS signal ratio (right 60 μm × 300 μm area/left 60 μm × 300 μm area) after hydrostimulation or one-side 1 nM zeatin treatment. Each circle represents the measurement from an individual root. Boxplots span the first to third quartiles of the data. Whiskers indicate minimum and maximum values. A line in the box represents the mean. “n” represents the number of roots used in this experiment. Scale bars represent 50 μm. One-way ANOVA with Tukey’s multiple comparison test was used for statistical analyses. $P < 0.001$.
