# Supplementary material for: Asymmetric distribution of cytokinins determines root hydrotropism in Arabidopsis thaliana
Source: Cell Res. 2019 Oct 10;29(12):984–93. doi: 10.1038/s41422-019-0239-3 (PMC6951336; doi:10.1038/s41422-019-0239-3)
Supplement: Supplementary file 23 — Supplementary information, Figure S23 [file 41422_2019_239_MOESM23_ESM.pdf]

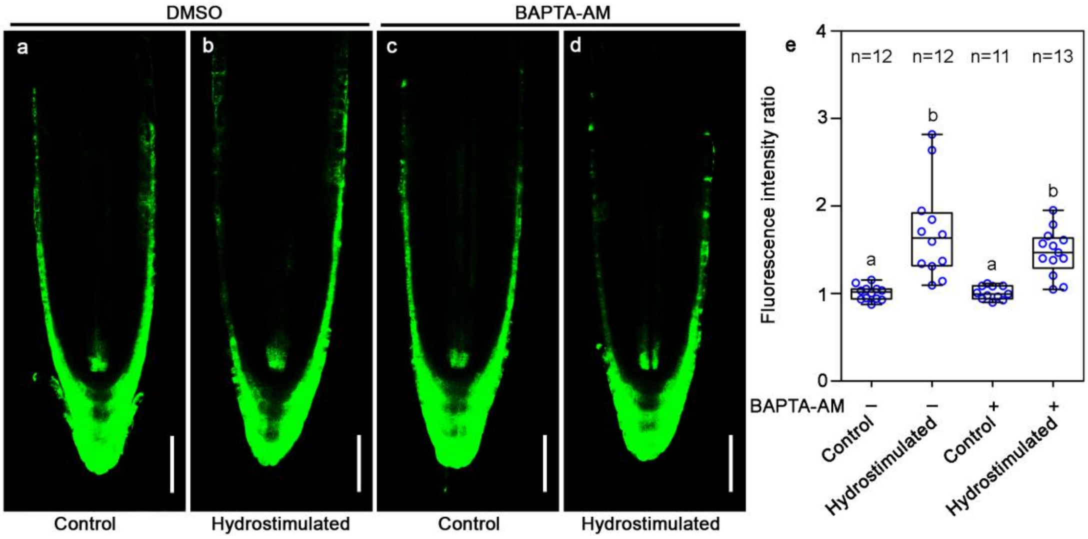

Supplementary information, Fig. S23 Ca^{2+} signaling is apparently not required for cytokinin asymmetric distribution or response during root hydrotropic response. a-b, Asymmetric GFP signal from *TCSn::GFP* plants can be observed after hydrostimulation treatment (**b**) with (**a**) as a control. **c-d**, Asymmetric GFP signal can still be seen after the treatment with hydrostimulation in *TCSn::GFP* and Ca^{2+} chelator BAPTA-AM in both sides of the split-agar media (**d**) with (**c**) as a control. **e**, GFP fluorescence ratio between right side and left side of lateral root caps (controls, with or without the treatment of BAPTA-AM), or between convex side and concave side of lateral root caps (hydrostimulated seedlings, with or without the treatment of BAPTA-AM) within a 200-µm meristematic zone starting from the quiescent center. Each circle represents the measurement from an individual root. Boxplots span the first to the third quartiles of the data. Whiskers indicate minimum and maximum values. A line in the box represents the mean. “n” represents the number of roots used in this experiment. Scale bars represent 50 µm. One-way ANOVA with Tukey’s multiple comparison test was used for statistical analyses. $P < 0.001$.
