# Supplementary material for: Asymmetric distribution of cytokinins determines root hydrotropism in Arabidopsis thaliana
Source: Cell Res. 2019 Oct 10;29(12):984–93. doi: 10.1038/s41422-019-0239-3 (PMC6951336; doi:10.1038/s41422-019-0239-3)
Supplement: Supplementary file 24 — Supplementary information, Figure S24 [file 41422_2019_239_MOESM24_ESM.pdf]

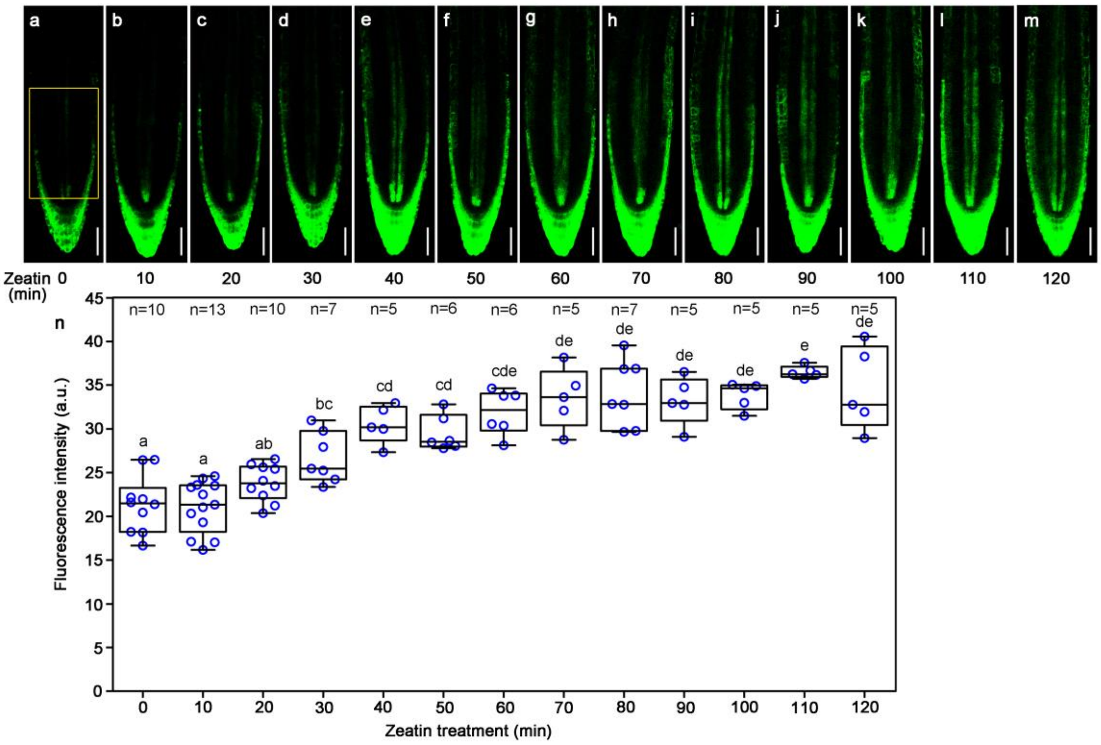

Supplementary information, Fig. S24 The GFP signal from the transgenic plants carrying *TCSn::GFP* can be rapidly induced by exogenous application of 100 nM zeatin. a-m, GFP signals at various time points after treatment with 100 nM zeatin. **n**, Measurements of fluorescence intensity using a Leica confocal software in an area of 200 μm × 120 μm in the root meristem zone (**a**). Each circle represents the measurement from an individual root. Boxplots span the first to the third quartiles of the data. Whiskers represent minimum and maximum values. A line in the box represents the mean. “n” represents the number of roots used in this experiment. Scale bars represent 50 μm. One-way ANOVA with Tukey’s multiple comparison test was used for statistical analyses. $P<0.01$.
