# Supplementary material for: Asymmetric distribution of cytokinins determines root hydrotropism in Arabidopsis thaliana
Source: Cell Res. 2019 Oct 10;29(12):984–93. doi: 10.1038/s41422-019-0239-3 (PMC6951336; doi:10.1038/s41422-019-0239-3)
Supplement: Supplementary file 25 — Supplementary information, Figure S25 [file 41422_2019_239_MOESM25_ESM.pdf]

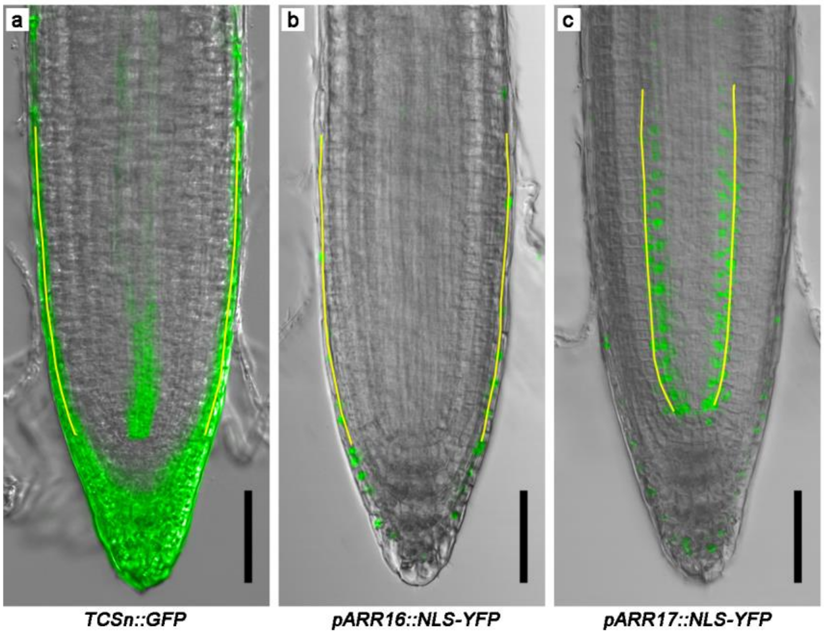

Supplementary information, Fig. S25 An approach used to measure the fluorescence intensity in single layer lateral-root-cap cells and endodermis. Root tip GFP or YFP signals from *TCSn::GFP* (**a**), *pARR16::NLS-YFP* (**b**), or *pARR17::NLS-YFP* (**c**) transgenic seedlings were measured as linear average intensity within a 200 µm distance above the quiescent center (as shown in figures) using a Leica confocal software quantification tool. Scale bars represent 50 µm.
